# Acute and Chronic Toxicity of Sediments Containing Platinum and Palladium on Freshwater Benthic Organisms *Chironomus riparius* and *Hyalella azteca*

**DOI:** 10.1002/jat.4933

**Published:** 2025-10-02

**Authors:** Alice Carle, Ludivine Preizal, Marc Amyot, Maikel Rosabal

**Affiliations:** ^1^ Département des Sciences Biologiques, Laboratoire d'Analyses Environnementales Université du Québec à Montréal Montréal Québec Canada; ^2^ Département de Sciences Biologiques, Complexe des Sciences Université de Montréal Montréal Québec Canada

**Keywords:** acute and chronic exposures, benthic organisms, platinum group elements, strategic metals, sediment toxicity

## Abstract

The expanding demand for platinum group elements (PGEs) in industrial and medical applications has led to their increasing accumulation in aquatic sediments. However, their ecological impacts remain poorly understood, particularly for sediment‐dwelling invertebrates. This study assessed the toxicity of platinum (Pt) and palladium (Pd) across a concentration range of 29–1214 μg·g^−1^ dw in two freshwater benthic species, *Chironomus riparius* (C) and *Hyalella azteca* (H), under both acute and chronic exposures. Pt was more toxic (LC_50_ in μg·g^−1^ dw; H: 289 ± 28; C: 84 ± 7) than Pd (H: 1192 ± 356; C: 209 ± 44) for acute survival, whereas Pd caused more pronounced sublethal effects on growth. Bioaccumulation patterns showed that *H. azteca* accumulated more Pt, whereas *C. riparius* retained more Pd. In *C. riparius* , chronic exposure to Pd impacted survival, emergence, and female adult weight, indicating developmental disruption. Compared with other sediment‐associated metals, Pd and Pt showed moderate to high toxicity: more toxic than uranium, nickel, arsenic, and molybdenum; comparable with copper; and less toxic than cadmium and lead. Although biological responses varied across metals, species, and endpoints, a consistent pattern of toxicity emerged. This study addresses a significant knowledge gap and reinforces the need to include PGEs in sediment quality guidelines. Although current environmental concentrations remain below toxicity thresholds, growing industrial use raises concerns for future ecological risk. Our findings support regulatory efforts by providing essential toxicity benchmarks and call for further research on mixture toxicity and mechanisms of action.

## Abstract 2

This study assessed the acute and chronic toxicity of platinum (Pt) and palladium (Pd) in contaminated sediments using *Chironomus riparius* and *Hyalella azteca* . Pt was more toxic for survival, whereas Pd strongly affected growth and emergence. Sensitivity and bioaccumulation patterns varied by species and metal. These findings highlight the differential effects of platinum group elements and support the development of sediment quality guidelines to protect freshwater ecosystems from increasing emissions linked to growing industrial PGE use.

## 1 Introduction

Platinum group elements (PGEs) are highly coveted metals because of their wide‐ranging applications in industrial processes, medical treatments, and the global energy transition (Azaroual et al. 2001; Balaram 2020). Their uses include catalytic converters (Twigg 2011), electronics, jewelry, dental implants, platinum‐based chemotherapy (Johnson Matthey 2024), and hydrogen technologies for low‐carbon energy (World Band Group 2020). As a result, PGE supply has become a critical issue for developing countries, where these elements are considered strategic for their economical, technological, and military development (MERN 2020). The escalating demand for PGEs has led to the diversification of extraction sources and an intensification of mining and recycling activities around the world, especially in leading producers of Pd and Pt such as South Africa, Russia, Zimbabwe, and Canada (USGS 2024). Such pressures have led to environmental contamination, particularly in urban and roadside dust sites (Schäfer and Puchelt 1998; Wei and Morrison 1994).

In aquatic environments, Pd and Pt tend to bind to suspended organic matter and accumulate in the sediments (CEAEQ 2022; HSDB 2024). Therefore, sediments represent a potentially dangerous source of Pd and Pt for freshwater benthic organisms. Elevated PGE contents have been observed in diverse countries and environmental matrices including sediments, soil, roadside dusts, and surface water near mining operations and along roads, underscoring substantial environmental contamination (Abdou et al. 2019; Bluteau et al. 2025; Wiseman et al. 2016). For example, in the Rhine sediments of Germany, Pt concentrations reached 31.2 μg·g^−1^ dw, indicating an enrichment of 10,000 times compared with natural levels of 0.5–4 ng·g^−1^ dw (Reith et al. 2014). In Toulon Bay (France), Pt in sediments increased from 12 to 16 ng·g^−1^ dw over the past 25 years, concomitantly with a 20‐fold rise in European Pt demand for the automotive catalyst industry (Abdou et al. 2019). Ice core measurements from Antarctic and Greenland revealed that current levels of PGEs are 40–120 times higher than 7000 years ago, and that human activities mainly account for the environmental increase of Pt, Pd, and rhodium (Rh) in recent decades (Barbante et al. 2001). Since the 1980s, the rise in environmental PGE levels has been largely attributed to their introduction in automotive catalytic converters (Barbante et al. 2001; Soyol‐Erdene et al. 2011).

Among the PGEs, Pd and Pt are the most soluble and mobile elements, which easily undergo a rapid transition (in less than 60 days) from particles to aqueous phases, increasing their potential for dispersion through water movement in aquatic environments (Fortin et al. 2011; Lustig et al. 1998). The bioaccumulation of Pd and Pt by various freshwater species in field studies—such as benthic crustaceans like *Asellus aquaticus* (Moldovan et al. 2001; Rauch and Morrison 1999) and *Gammarus* spp. (Haus et al. 2007), mollusks like *Corbicula* spp. (Ruchter 2012) and *Dreissena polymorpha* (Zimmermann et al. 2002), and fish such as *Barbus barbus* (Sures et al. 2015)—has long supported the view that these metals can enter aquatic food webs (Zereini and Wiseman 2015). However, recent work by Bluteau et al. (2025) challenges this assumption by showing low bioaccumulation and poor trophic transfer of PGEs in a predator–prey model ( *Chaoborus americanus* exposed to PGE‐contaminated *Daphnia magna* ), supported by low detection of PGEs in wild organisms and rapid excretion rates. This study highlights that bioaccumulation remains a key parameter to assess when evaluating the ecotoxicological effects of PGEs.

Both Pd and Pt exhibit significant toxicity in aquatic ecosystems. Exposure of *H. azteca* to freshwater contaminated with either Pt or Pd revealed that Pt was more toxic than Pd, with a 7‐day median lethal concentration (LC_50_) value of 131 μg·L^−1^ for Pt and above 1000 μg·L^−1^ for Pd (Borgmann et al. 2005). Inversely, a 24‐day exposure of *A. aquaticus* showed greater mortality for Pd than Pt at 500 μg·L^−1^, with a dead rate of 47% for Pd and 34% for Pt (Moldovan et al. 2001). So far, results regarding the sublethal and lethal toxicity of Pd and Pt in contaminated water have been reported, but studies on sediment have been restricted to terrestrial organisms (Havelkova et al. 2014). In addition, a recent literature review (CEAEQ 2022) highlights a notable lack of recent studies, particularly from the 2020s, addressing environmental concentrations of Pd and Pt or their toxicity. To our knowledge, no studies have investigated the toxicity of PGEs on aquatic benthic organisms exposed to contaminated sediments. The use of benthic invertebrates such as *C. riparius* (Meigen 1803) and *H. azteca* (Saussure 1858) is pertinent since these organisms live in close interaction with sediments, and they have been widely used to study both acute and chronic impacts of various trace metals on aquatic organisms (Ingersoll et al. 1996). Considering the increasing PGE release and high concentrations expected to be found in the environment soon, particularly in aquatic sediments (Barbante et al. 2001), it is timely to generate toxicological data on PGEs in sediments to characterize their toxicity to benthic organisms after acute and chronic exposures. Although most studies on Pd and Pt toxicity have focused on the water column (Boukhari et al. 2025; Wren and Gagnon 2014; Zimmermann et al. 2015), little is known about their effects in sediment, particularly under chronic exposure. This gap hinders risk assessment for benthic invertebrates exposed to these emerging contaminants.

To address these gaps in understanding the toxicological impacts of PGEs, the present research aims to evaluate the acute and chronic toxicities of Pd and Pt on *C. riparius* and *H. azteca* . To gain more insights into PGE toxicity, we also determine the Pd and Pt bioaccumulation in both organisms exposed to acute conditions. By comparing results obtained between species and metal, and with environmental and toxicological data of other metals, we aim to provide a comprehensive understanding of the relative risks posed by these two elements in aquatic environments.

## 2 Methods

### 2.1 Animal Cultures

*H. azteca* and *C. riparius* were purchased from Aquatic Research Organisms Inc., and their culture was maintained at the animal facility of the *Université du Québec at Montreal* (UQAM, QC, Canada). Both organisms were acclimated to laboratory conditions for at least 1 month prior to use. They were cultured in an environmental chamber with a photoperiod of 16‐h light, under a temperature of 23°C ± 1°C for *H. azteca* and 20°C ± 1°C for *C. riparius* . *H. azteca* were cultured in 4‐L glass beakers containing reconstituted water, and *C. riparius* in a 20‐L tank containing artificial sand (CaribSea Moonlight Sand, Super Naturals) and reconstituted water. For *H. azteca* , the reconstituted water was prepared from distilled water according to standardized protocols outlined by ISO (2013), and Environment Canada (2017). It contained CaCl_2_ (110.98), NaHCO_3_ (84.01), MgSO_4_ (30.09), KCl (3.73), and NaBr (1.03) mg·L^−1^, with a water hardness between 120 and 140 mg·L^−1^ as CaCO_3_. For *C. riparius* , the reconstituted water consisted of CaSO_4_·2H_2_O (0.05), CaCl_2_·2H_2_O (0.05), MgSO_4_·7H_2_O (0.03), NaHCO_3_ (0.096), and KCl (0.004) g·L^−1^, with a water hardness between 90 and 100 mg·L^−1^ as CaCO_3_. Cultures of *H. azteca* were fed with Hiraki spirulina pellets, and cultures of *C. riparius* with Tetramin fish food flakes once a week. Culture water was changed at least once a week.

### 2.2 Toxicological Testing

Physicochemical characteristics of this sediment revealed no significant metal contamination (Table S1). Palladium (II) chloride and platinum (II) chloride salts were purchased from Sigma Aldrich (PdCl_2_ and PtCl_2_). Pd and Pt salts were stirred with the sediment using a spatula until homogeneity, as described in Environment Canada (1995). For dose–response experiments, six different Pd or Pd nominal concentrations were tested, ranging from 50 to 2000 μg·g^−1^ dw of dry sediment (dw), each including six replicates. To ensure that any observed toxic effects were only due to the metals and not to differences in chloride concentration added with the metal salts, potassium chloride (KCl) salt levels (Sigma Aldrich) were adjusted to make sure each condition contains the same amount of chloride (i.e., the Cl level of the highest metal content condition). Humidity was also adjusted to the same level in all conditions to allow homogenization and standardize the mixing procedure and conditions. Contaminated sediment consisted of one‐half of natural sediment collected from Chaudière River (GPS 45°43′14.9″N 70°44′00.6″W, Quebec, Canada) and stored at −20°C before use and one‐half of artificial sand (CaribSea). Such a combination of sediments was chosen to maintain a pH above 7 for all treatments and to mimic natural conditions by providing organic matter from field sediments instead of artificial organic matter, such as α‐cellulose, commonly added in toxicity tests using artificial sediment alone (Carney Almroth et al. 2023; Lacey et al. 1999). After one night, the sediments were distributed into six exposure beakers. To achieve a stable pH above 7, which is necessary for organism survival, the sediments underwent a 7‐day equilibration period, as detailed in Supporting Information S1. At the start of the experiment, two replicates were used to quantify the initial metal concentrations in the sediment, whereas the remaining four replicates were used for organism exposure. Parameters such as temperature, pH, oxygen, and conductivity were monitored throughout the experiments using a MultiLab 4010‐3W (YSI), and nitrites, nitrate, and ammonium levels were evaluated with freshwater tests (API).

#### 2.2.1 *H. azteca* Toxicity Testing

According to ISO 16303 (2013) guideline, acute 14‐day tests were performed with sediment enriched with nominal concentrations of Pd: 100, 200, 400, 800, 1600, and 2000 μg·g^−1^ dw or Pt: 100, 200, 400, 800, 1600, and 2000 μg·g^−1^ dw. Juveniles of 5‐ to 7‐day‐old were exposed by a group of 10 individuals in each of the four replicates per testing condition.

#### 2.2.2 *C. riparius* Toxicity Testing

According to OECD (2004) guideline, acute 10‐ and chronic 28‐day tests were performed with sediment enriched with nominal concentrations of Pd: 100, 200, 400, 800, 1600, and 2000 μg·g^−1^ dw or Pt: 50, 100, 150, 200, 250, and 500 μg·g^−1^ dw. Two‐ to Three‐day‐old larvae of *C. riparius* were also exposed by groups of 10 individuals in each of the four replicates per testing condition. The combination of acute (10‐day) and chronic (28‐day) exposures allowed us to detect both immediate and delayed effects of metal exposure, providing a more comprehensive assessment of toxicity across ecologically relevant timescales.

To maintain stable physicochemical parameters suitable for organism survival, water was renewed mid‐exposure (on Day 7 for *H. azteca* and Day 5 for *C. riparius* ; Figure S1). Water changes during the exposure period ensured stable physicochemical parameters, with no significant variations during the exposure tests, as shown in Table S2. Both exposed organisms were fed three times per week with a fish food solution of TetraMin flakes (Tetra) at 6.3 mg per test chamber for *H. azteca* (ISO 16303 2013) and with an increased amount of TetraMin flakes for *C. riparius* (OECD 2004). Further details on parameter measurements and quality control for toxicity tests are provided in Supporting Information S2 and S3.

### 2.3 Metal Measurements

Sediment samples taken at the beginning and end of each test to estimate metal content were freeze‐dried, and 0.5 g dw of each sample was acidified with 10 mL of metal‐trace HNO_3_ (Fisher Scientific) and 2 mL of HCl (Fisher Scientific), digested during 45 min (rise/hold/ set: 10 min/25 min/10 min) at 185°C in a Multiwave 5000 digestion platform (Anton Paar) and subsequently analyzed using an inductively coupled plasma‐mass spectrometer triple quadrupole (8990, ICP‐MS/MS, Agilent, USA) at the *Laboratoire d'Analyses Environnementales* of *Université du Québec à Montréal* (UQAM).

After the exposure period, individuals underwent a depuration step to remove metal from the digestive tract. The organisms were placed under the same conditions as exposures for a period of 48 h for *H. azteca* and 72 h for *C. riparius* in 250‐mL jars of reconstituted water. Then, they were rinsed to remove metals from the organism's surface to better estimate metal internalization (Geffard et al. 2010). This rinsing procedure consisted of 5 min in reconstituted water, 10 min in a 10‐mM solution of ethylenediaminetetraacetic acid (EDTA; Bio Basic Canada Inc), and 5 min with reconstituted water. Samples were then stored at −20°C until analysis. Frozen organisms were freeze‐dried prior to analysis. At the time of analysis, the dry weight of the organisms was determined before digestion. Further details on the digestion protocol and Pd and Pt analysis quality controls via ICP‐MS/MS are provided in Supporting Information S4 and S5 and Table S3 (recovery percentage) and Table S4 (details on nominal and measured metal concentrations). High metal concentrations were used in this study, as no effects were observed at lower levels in preliminary tests. Reaching observable toxicity was necessary to establish relevant toxicity thresholds.

### 2.4 Data Treatment and Statistical Analyses

#### 2.4.1 Acute Exposure Calculations

To assess the effect of Pd and Pt on growth, the specific growth rate (SGR) was calculated (Crémazy et al. 2018) for each replicate (Equation 1):

(1) $$\mathrm{SGR}=\frac{\ln\left(w_{\text{final}}\right)\times \ln\left(w_{\text{initial}}\right)}{t}\;\left(\text{in}\;\mathrm{day}-1\right)$$

where $\left(w_{\text{final}}\right)$ and $\left(w_{\text{initial}}\right)$ are respectively the initial and the final length means and *t* is the exposure time. To make a comparison between treatments, the relative growth rate (RGR) was calculated (Equation 2):

(2) $$\mathrm{RGR}=\frac{\mathrm{SGR}}{{\mathrm{SGR}}_{\text{control}}}\;\left(\text{in}\%\right)$$

The SGR was calculated using Equation (1) for each replicate, whereas SGR_control_ represents the mean of both SGR calculated for the negative and KCl controls, as no significant difference between both was found (Supporting Information S3).

The survival rate was estimated by dividing the number of alive individuals at the end by the initial number before exposure.

#### 2.4.2 Chronic Exposure Calculations

Survival was calculated using the same method as for the acute tests. The emergence time represents the number of days necessary for the larvae to reach the adult stage. The analyses of adult emergence time were based on data from all emerged adults—viable, nonflying, and dead—whereas the analyses of adult weight only included data from viable flying adults (Monteiro et al. 2019). Adults found on the water were excluded, as their presence of water on their surface could have increased weight and biased the results. The sex ratio was quantified according to the male–female ratio (Khosrovyan and Kahru 2020).

#### 2.4.3 Modeling and Toxicity Parameters

For modeling the concentration–response curves and to determine the lethal concentrations (LC_x_), a Log‐Logistic model with 4 parameters (LL4) with the end‐of‐curve parameter set to 0 was applied using the drc() package (Ritz and Streibig 2014) of RStudio. Among all tested models (LL2, LL3, Weibull 1, Weibull 2, Brain‐Cousens 4, Brain‐Cousens 5), the LL4 model provided the best fit to the data and was therefore selected for estimating LC_50_ values. Analysis of the fit of the data for the linear model (*R* ^2^ and *p*‐value = *p*) was also carried out in RStudio using the lm() function in the stats package. Four toxicity parameters were estimated in the analyses: LC_20_, LC_50_, LOEC, and NOEC. LC_20_ and LC_50_ represent the lethal concentration causing 20% and 50% mortality, respectively, whereas LOEC (lowest observed effect concentration) and NOEC (no observed effect concentration) correspond to the lowest concentrations at which a significant effect was or was not observed compared with the control. These endpoints provide complementary insights into the toxicity of Pd and Pt, as well as the sensitivity of the test organisms.

#### 2.4.4 Statistical Analysis

Statistical analyses were performed using RStudio software. Since the assumptions of normality and homoscedasticity were not met, as indicated by Shapiro–Wilk's and Bartlett's tests, respectively, significant differences between the tested conditions and the controls were tested using a Kruskal–Wallis chi‐squared test followed by Dunn's multiple comparison test with Bonferroni adjustment (*p* < 0.05). Significant differences are represented by an asterisk (*) in the figures. All graphical representations using an LL4 or a linear model presented in this study demonstrated statistically significant *p*‐values (*p*), indicating a strong fit of the data to the proposed model. For chronic weight of *C. riparius* adults, the difference between males and females of each condition was tested using a Wilcoxon–Mann–Whitney test (*p* < 0.05).

## 3 Results

### 3.1 Acute Toxicity of Pd and Pt on *H. azteca* and *C. riparius*

#### 3.1.1 Acute Survival

**FIGURE 1:**
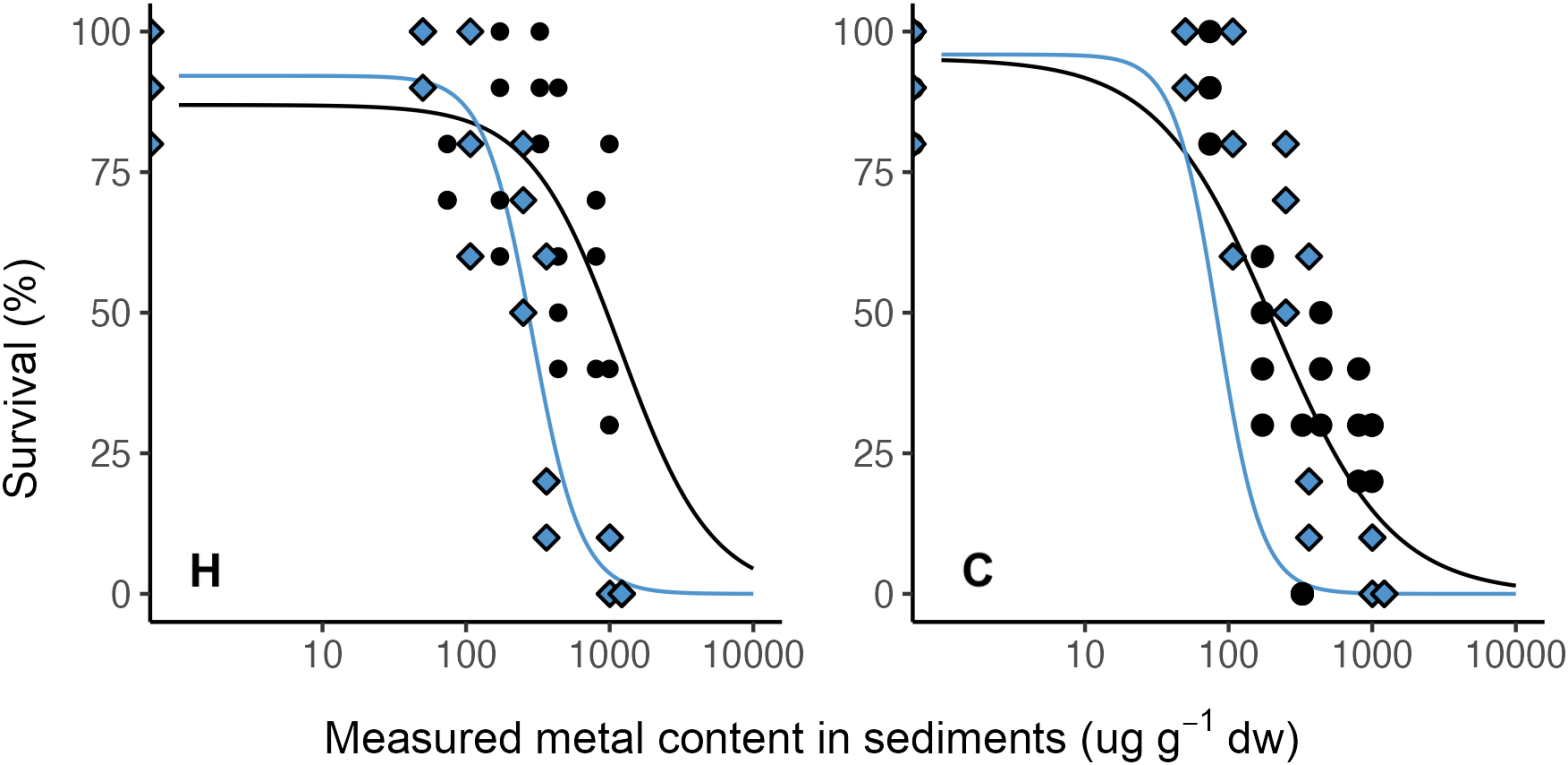
Concentration–response curves for survival (%) as a function of the measured content (μg_metal_·g_sed_^−1^ dw) of Pd (black points) and Pt (blue rhombuses) following exposure of *H. azteca* (H) and *C. riparius* (C) to contaminated sediments. Asterisks (*) denote significant differences compared with control conditions (*p* ≤ 0.05, *n* = 4).

Sediments contaminated with Pd or Pt had adverse effects on the survival of the two exposed organisms, *H. azteca* and *C. riparius* , compared with control conditions (Figures 1 and S2). When *H. azteca* and *C. riparius* were exposed to Pt‐contaminated sediments, the LC_20_ and LC_50_ values were found to be significantly (*p* < 0.05) lower than the values obtained with Pd (except LC_20_ for *C. riparius* ; Table S5). When comparing LOEC values (Figure S2), Pt induced effects at lower concentrations than Pd on *C. riparius* , whereas both metals triggered significant effects at comparable concentrations on *H. azteca* (995 and 1002 μg·g^−1^, respectively). We observed that the benthic larvae *C. riparius* exhibited a greater sensitivity to sediments contaminated with Pd or Pt than the epibenthic crustacean *H. azteca* , where LC_20_, LC_50_, NOEC, and LOEC values were consistently lower for *C. riparius* than for the amphipods. Based on the LC_20_ and LC_50_ values for both metals, the sensitivity of *C. riparius* is eight times (LC_20_ for Pd) and three times (LC_20_ for Pt) higher than that of *H. azteca* .

#### 3.1.2 Acute Relative Growth Rate

**FIGURE 2:**
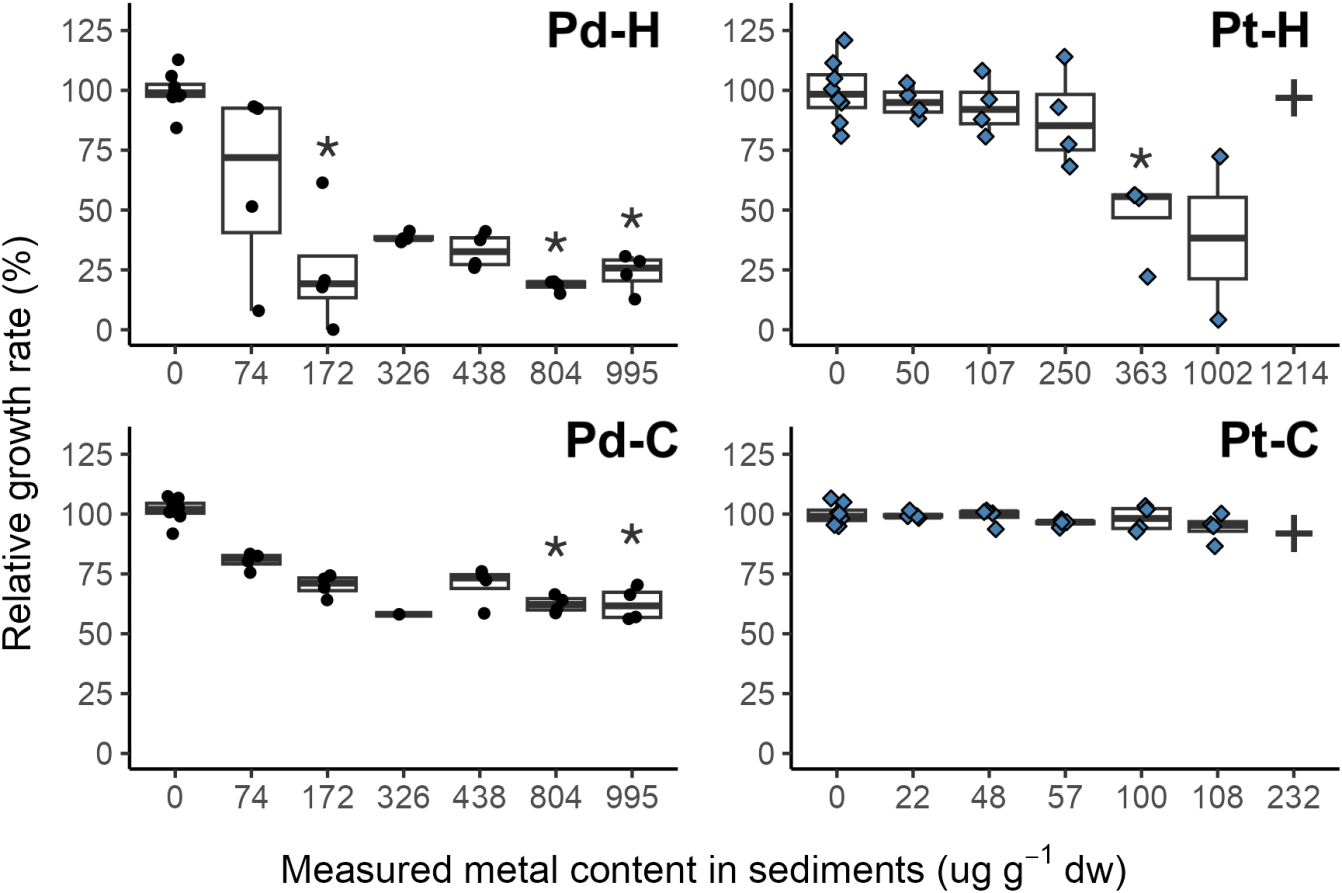
Relative growth rate (%) as a function of the measured content (μg_metal_·g_sed_^−1^ dw) of Pd (black points) and Pt (blue rhombuses) following exposure of *H. azteca* (H) and *C. riparius* (C). The cross symbol (✚) denotes the absence of data due to complete mortality of the exposed organisms. Asterisks (*) denote significant differences compared with control condition (*p* ≤ 0.05, *n* = 4).

The growth of *H. azteca* and *C. riparius* was also negatively affected by Pd‐contaminated sediments compared with growth obtained under control conditions (Figure 2). According to LOEC values, Pd was at least 2 times more toxic than Pt on both organisms (Table S6). Pd caused more inhibition than Pt in *H. azteca* , where LOEC and NOEC values were 3.3‐fold and 2.1‐fold lower than the respective values for Pt. In contrast to *H. azteca* , the LOEC and NOEC levels found in *C. riparius* for Pd were higher than those values reported for Pt. Note that after the exposure to Pt at 232 μg·g^−1^ dw, all larvae died, which implies a NOEC and LOEC of 108 and 232 μg·g^−1^ dw, respectively.

Additionally, relationship analysis of the RGR (in %) as a function of metal content in sediment revealed a significant linear decrease (*p* < 0.05) in growth as metal content increased and that for both metals and in both organisms (Figure S3). Moreover, a significant linear correlation was also observed between acute RGR (%) and survival (%) for all the conditions, except for *C. riparius* exposed to Pt due to larval mortality at low content (Figure S4).

#### 3.1.3 Acute Bioaccumulation

**FIGURE 3:**
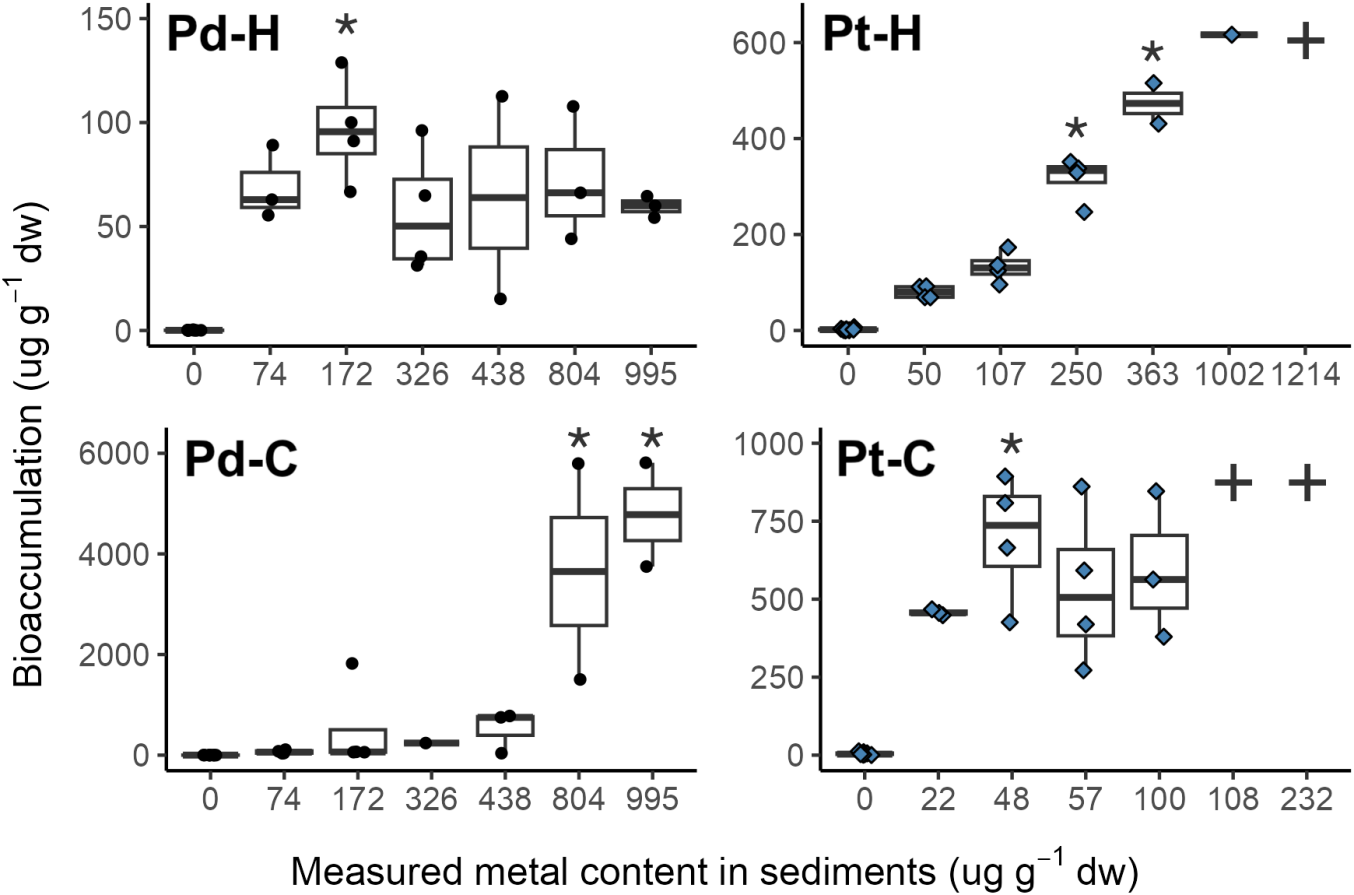
Bioaccumulation (μg_metal_·g_organism_^−1^ dw) as a function of the measured content (μg_metal_·g_sed_^−1^ dw) of Pd (black points) and Pt (blue rhombuses) following exposure of *H. azteca* (H) and *C. riparius* (C), respectively. The cross symbol (✚) denotes the absence of data at this concentration due to complete mortality of the exposed organisms. Asterisks (*) denote significant differences compared with control conditions (*p* ≤ 0.05, *n* = 4).

With regard to bioaccumulation data, both *H. azteca* and *C. riparius* significantly (*p* < 0.05) accumulated Pd and Pt after acute exposures to sediment contaminated (Figure 3). Toxicological parameters for bioaccumulation (NOEC; LOEC) are shown in Table S7. The LOECs indicate that Pd induced effects at levels 2.1 times lower than Pt in *H. azteca* , whereas in *C. riparius* , Pt caused significant effects at concentrations 3.5 times lower than those of Pd. Moreover, *H. azteca* bioaccumulated Pd for a maximum at 129 μg·g^−1^ dw, in contrast to *C. riparius* that accumulated 45 times more Pd (5812 μg·g^−1^ dw) for tested metal concentrations. The maximum capacity of *H. azteca* to bioaccumulate Pt was 685 μg·g^−1^ dw for tested metal concentrations. For *C. riparius* , the maximum capacity to accumulate Pt was 893 μg·g^−1^ dw for tested metal concentrations ranging from 22 to 232 μg·g^−1^ dw.

Comparison of the fit of bioaccumulation data using various models (log‐logistic, Michaelis–Menten, and fitted linear model) indicated that the linear model provided the best fit relative to sediment metal contents (Figure S5). Also, significant linear relationships were obtained between the whole‐body metal content and the measured metal levels in sediments for both metals as well as both animal models. Examining the links between bioaccumulation and survival or growth (Figure S6) in both benthic organisms, significant linear correlations (*p* < 0.05) were also revealed (except for Pd [*n* = 4] for *H. azteca* [*p* = 0.1] and Pt for *C. riparius* [*p* = 0.3]). For all these relationships, a decrease in survival and growth rate was observed as whole‐body metal content increased.

In summary, acute exposure to Pd‐ and Pt‐contaminated sediments caused significant toxicity in *H. azteca* and *C. riparius* , with *C. riparius* showing greater sensitivity overall. Despite Pt inducing the sharpest decline in survival (Figure 1 and Table S5), Pd exposure resulted in a greater number of concentrations with statistically significant effects (Figure 2 and Table S6). Bioaccumulation was metal‐ and species‐dependent (Figure 3 and Table S7), with significant correlations observed between internal concentrations and both survival and growth endpoints.

### 3.2 Chronic Toxicity of Pd and Pt on *C. riparius*

#### 3.2.1 Acute Survival

**FIGURE 4:**
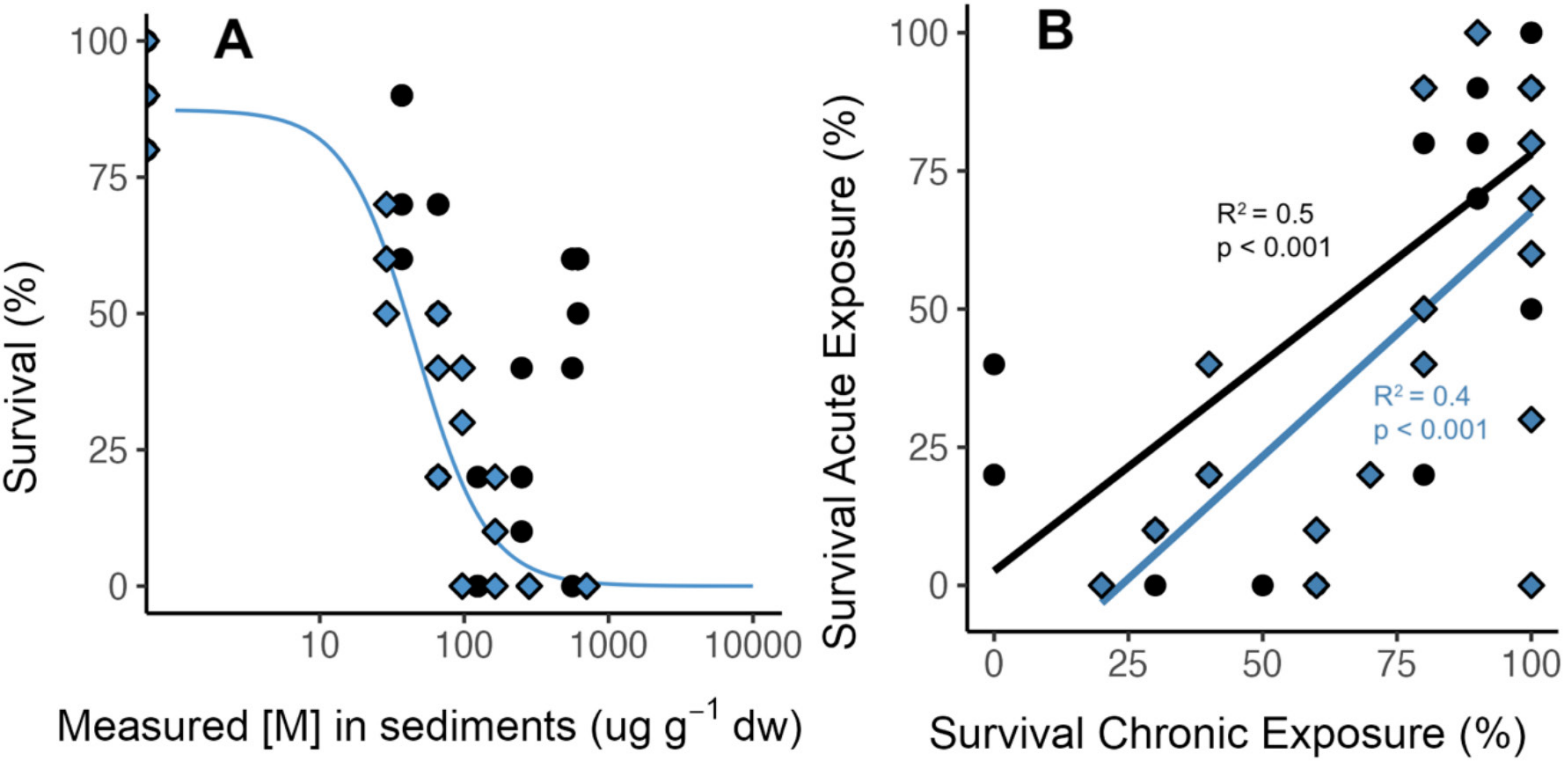
(A) Concentration–response curve for survival (%) as a function of the measured content (μg_metal_·g_sed_^−1^ dw) of Pd (black points) and Pt (blue rhombuses). (B) Linear regression curves for acute survival (%) as function of chronic survival. Adjusted *R*‐squared and *p*‐value estimated are shown.

Since *C. riparius* was the most sensitive organism in terms of survival in response to acute exposures, chronic assays were carried out on this species. The chronic survival of *C. riparius* larvae was negatively impacted by exposure to both Pd‐ and Pt‐contaminated sediments. Toxicological parameters (LC_20_; LC_50_) are shown in Table S8. Chronic exposures to Pd‐contaminated sediments exhibited higher toxicity on survival than Pt‐contaminated sediments when looking at the LC_20_ and LC_50_ values, which is consistent with the LOEC values (Figure S7). Moreover, a linear regression (*p* < 0.05) of chronic and acute survival was observed (Figure 4B), demonstrating consistency between acute and chronic survival despite different metal contents in sediments.

#### 3.2.2 Chronic Sex Ratio

Whether for Pd (*p* = 0.4) or Pt (*p* = 3), no significant effect of metal content on the sex ratio of emerged adults was observed (Figure S8). These results suggest that there was no significant difference in the number of females and males between uncontaminated and contaminated sediment exposures.

#### 3.2.3 Chronic Emergence Time

Emergence time (in days) of *C. riparius* adults exposed to Pd was significantly (*p* < 0.05) higher compared with the control for all tested conditions (from 29 to 705 μg·g^−1^ dw; Figure S9), indicating a negative effect of Pd on the emergence capacity of the organisms.

In contrast, no significant difference was found for Pt, which caused rapid and complete mortality of individuals after 164 μg·g^−1^ dw. Chronic survival (Figures 4A and S7) and emergence time (Figure S9) follow the same trend as results obtained for survival (Figures 1 and S2) and growth (Figures 2 and S3) under acute exposures, respectively.

#### 3.2.4 Chronic Weight

At the flying insect stage of *C. riparius* , a Kruskal–Wallis test revealed that adult weight was not significantly influenced by the concentration of metals in the sediments (Figure S10). However, pairwise comparisons using the Wilcoxon–Mann–Whitney test between males and females within each exposure condition highlighted that females were significantly heavier than males only under the lowest (37 μg·g^−1^ dw) and the highest tested concentrations (615 μg·g^−1^ dw) of Pd, as well as the first two tested concentrations of Pt (29 and 66 μg·g^−1^ dw). Interestingly, this significant sexual dimorphism was also observed in the control group, suggesting that under normal conditions, females naturally reach higher biomass than males. Yet, under intermediate and higher metal concentrations, this difference tended to diminish or disappear. This pattern suggests that exposure to Pd and Pt may attenuate the natural sexual dimorphism in weight by affecting female growth more severely than that of males. Overall, chronic effects are aligned with acute patterns. Chronic exposures to Pd and Pt reduced *C. riparius* survival, with Pd being more toxic. Pd delayed emergence; Pt caused early mortality. No significant effects were found on sex ratio or weight, but metal exposure reduced female weight.

## 4 Discussion

### 4.1 Importance of Short‐ and Long‐Term Toxicological Data in Sediments

Our findings fill a key knowledge gap by providing the first sediment‐based as well as chronic toxicity data for Pd and Pt in benthic invertebrates, supporting the need to complement water‐only studies with sediment‐focused assessments (CEAEQ 2022; Cobelo‐García et al. 2015). This is particularly critical given the rising accumulation of these metals in aquatic sediments and the limited capacity of short‐term aqueous tests to reveal sublethal and lethal effects (Borgmann et al. 2005; Lüderwald et al. 2016). Table S9 summarizes the LC_50_ and EC_50_ values reported in the literature for Pd and Pt across terrestrial and aquatic invertebrates and highlights how our findings complement and expand current knowledge, especially by providing unique sediment‐based toxicity data.

### 4.2 Pd and Pt Induce Lethal and Sublethal Toxicity

This study yields the evidence that both Pd and Pt significantly affect the survival, growth, emergence, and weight of freshwater benthic invertebrates when present in sediments. Notably, Pd showed broader effects across endpoints and concentrations on both species, whereas Pt caused sharper but more isolated toxicity. These findings underline the distinct and complementary modes of action of these metals under sediment exposure conditions, emphasizing the need to include both in sediment quality assessments. In this study, Pt was more toxic than Pd on acute survival, which is consistent with results reported for the survival of *H. azteca* exposed to Pd or Pt in hard water for 168 h (Borgmann et al. 2005). However, as observed for growth, the opposite was found in the survival and mobility of *Daphnia magna* after 24, 48, 96, and 144 h (Zimmermann et al. 2017), where Pd was shown to be more toxic than Pt. Going beyond previous water‐based studies, this research showed that Pd and Pt exert species‐ and endpoint‐specific toxicity in sediments. Our results show strong and consistent linear correlations between growth rate (Figure S3) and metal content, where the growth rate tended to increase in conditions where more organisms survived (Figure S3). These relationships highlight the coherence and interdependence between survival and growth endpoints, suggesting that growth responses are closely tied to survival rates.

### 4.3 Metal Bioaccumulation

Metal bioaccumulation is currently one of the best indicators of exposure and bioavailability of environmental pollutants (Luoma and Rainbow 2005; Marsden and Rainbow 2004). Commonly used in ecotoxicological studies, it can be used to diagnose the toxicity of metals and their ecological impact (Luoma and Rainbow 2005). However, limited information is available on Pt and Pd bioaccumulation in benthic invertebrates used for toxicological tests. In this study, the bioaccumulation of Pd and Pt was evaluated despite challenges with organism integrity at the highest exposure levels. Although some replicates at these concentrations were not suitable for analysis, as the organisms were severely decomposed or physically deteriorated, this prevented accurate analysis. Data from the remaining samples after the depuration step were used to assess metal accumulation in organisms exposed to Pd or Pt (Figures 3 and S5).

Our results showed that *H. azteca* accumulated four times more Pt than Pd at equal metal concentrations in the media, yet without exhibiting toxic effects, whereas *C. riparius* accumulated twice as much Pd as Pt despite being exposed to Pt concentrations four times lower than those of Pd (Figure 3). Consequently, no clear trend emerged regarding the preferential accumulation of Pt or Pd in either of the benthic species, suggesting that metal uptake may be species‐ or context‐dependent. The predominant accumulation of Pt over Pd within the organism has already been documented in the polychaeta *Arenicola marina* (French and Turner 2008). Although the external content of Pd was higher than that of Pt for *A. marina* , the assimilation efficiency was estimated at 10% for Pt and 1% for Pd. This may be due to the capacity of Pd to bind fulvic compounds, whereas Pt binds preferentially to the biologically available fraction of humic matter (Sures et al. 2002).

Our findings in *H. azteca* , showing higher internal Pt concentrations without observable effects, may also reflect this preferential binding to less bioavailable forms, limiting its toxicity (Balaram 2020; Fortin et al. 2011). In contrast to these results, an important accumulation of Pd over Pt, reported in our study in *C. riparius* , was also previously observed in algae (Cosden et al. 2003), terrestrial plants (Schäfer and Puchelt 1998), and aquatic plants (Bonanno and Pavone 2015). Indeed, a higher accumulation of Pd compared with Pt has also been reported in aquatic invertebrates, such as the crayfish *Orconectes virilis* (Wren and Gagnon 2014), the freshwater epibenthic isopod *Asellus aquaticus* (Moldovan et al. 2001), and the zebra mussel *Dreissena polymorpha* (Sures et al. 2002). It has been considered that the high mobility of Pd may increase its bioavailability for benthic organisms (Leopold et al. 2017). Additional details on the parameters influencing metal uptake in organisms are provided in Supporting Information S6. Altogether, the results highlight that bioaccumulation is governed by multiple biological and environmental factors, which must be considered when investigating metal bioaccumulation patterns and their potential toxicity.

### 4.4 Species‐Specific Sensitivity and Endpoint‐Specific Toxicity

Striking interspecies differences emerged under acute Pd and Pt exposure, revealing distinct vulnerability profiles between *C. riparius* and *H. azteca* . *H. azteca* exhibited a higher sensitivity to Pd, with a growth LOEC at least four times lower than that observed for *C. riparius* (Table S6). For Pt, no LOEC could be established for *C. riparius* due to mortality preceding significant effects on growth. Although *C. riparius* appeared more sensitive in terms of survival, *H. azteca* exhibited greater susceptibility through growth‐related endpoints. Together, these findings underline the greater sensitivity of growth as an endpoint in *H. azteca* , making it a valuable indicator in sediment toxicity assessment for this species.

Moreover, our results clearly show that *C. riparius* bioaccumulated significantly higher levels of both Pt and Pd compared with *H. azteca* , suggesting species‐specific differences in metal uptake. These differences are likely linked to their contrasting ecological niches and feeding behaviors. Although *H. azteca* is an epibenthic species that feeds partly on suspended organic matter in the water column, *C. riparius* larvae inhabit and feed directly within the sediment, accessing organic material and metals more readily from sediment and porewater. This sediment‐dwelling lifestyle, combined with a diet more tightly linked to sediment particles, likely enhances metal exposure and assimilation efficiency in *C. riparius* . These findings underscore the importance of species ecology in interpreting bioaccumulation patterns and suggest that benthic invertebrates with different feeding habits may exhibit distinct sensitivities to sediment‐bound metals. Further investigations using subcellular partitioning, along with metallomic and proteomic approaches, could provide valuable insights into the cellular pathways and mechanisms driving Pt and Pd toxicity in the organisms (Martins et al. 2019; Racine 2016).

The toxicity of contaminated sediments to *H. azteca* and *C. riparius* has previously been assessed for several metals with physicochemical properties similar to Pd and Pt across various exposure durations (Table S7), providing a relevant basis for comparison with the results of the present study. For copper (Cu) as an example, Roman et al. (2007) found that the growth (EC_50_) of *H. azteca* was higher than that of *C. riparius* , demonstrating that the latter was the more sensitive species. Interestingly, the reverse was observed for survival, for which *H. azteca* became more sensitive than *C. riparius* . As observed in our study, the sensitivity of either species changes depending on the endpoints analyzed and the species exposed. Our results and those of Roman et al. (2007) highlight the importance of considering multiple metals, species, and endpoints when assessing the ecological risks of metal contaminants.

This species‐ and endpoint‐specific toxicity likely results from a combination of biological, ecological, and experimental factors. Differences in exposure duration, based on guideline recommendations (14 days for *H. azteca* vs. 10 days for *C. riparius* ), may partly explain variations in sensitivity. Moreover, the two species occupy distinct ecological niches ( *H. azteca* being epibenthic and *C. riparius* being sediment‐dwelling), leading to different exposure routes and metal uptake dynamics.

At the mechanistic level, Pd and Pt are group B elements that can form strong bonds with sulfur‐containing biomolecules, potentially disrupting essential cellular functions such as enzyme activity and redox balance (Mason and Jenkins 1995). Additionally, bioaccumulation patterns in *O. virilis* exposed to PGE mixtures revealed a synergistic increase in Pt uptake, particularly in the hepatopancreas, accompanied by reduced Pd accumulation—likely due to competitive use of membrane transporters (Wren and Gagnon 2014). Such interactions suggest that bioavailability and transport specificity strongly influence toxicity. Moreover, Pt is widely used in medical applications due to its cytotoxic effects on cell division processes, including DNA replication and apoptosis induction (CEAEQ 2022), which enhance cellular toxicity.

These combined factors may explain why one species appears more sensitive to a given endpoint (e.g., survival in *C. riparius* ), whereas the other is more affected in terms of growth (e.g., *H. azteca* ).

### 4.5 Long‐Term Toxicity

*C. riparius* emerged as the most vulnerable species in acute scenarios, highlighting its relevance as a model organism for chronic toxicity assessments. This justified its inclusion in extended exposure assays. As previously reported, tolerance can increase with larval development, emphasizing the importance of comparing both acute and chronic responses (Gauss et al. 1985; Pascoe et al. 1989; Powlesland and George 1986; Williams et al. 1986). Chronic survival of *C. riparius* larvae was significantly reduced by exposure to both Pd‐ and Pt‐contaminated sediments, with Pd showing higher toxicity based on LC_20_ and LC_50_ values (Table S8). Interestingly, the significant linear regression between acute and chronic survival (Figure 4B) highlights the consistency of toxic effects across exposure despite differences in duration and tested metal concentrations.

The significant increase in adult emergence time observed across all six tested Pd concentrations (Figure S9), whereas only two concentrations significantly affected survival (Figure S7), suggests that sublethal endpoints such as development delays are more sensitive indicators of toxicity than survival alone. This heightened sensitivity of growth compared with survival has been documented in *C. riparius* exposed to metals such as nickel (Powlesland and George 1986) and other chemicals in water such as copper and pesticides (Taylor et al. 1991). Similarly, studies with *Chironomus tentans* larvae exposed to contaminated sediments have demonstrated that larval growth is a biologically relevant, sensitive endpoint and that survival alone does not accurately describe the potential long‐term effects of sediment toxicity (Call et al. 1993; Liber et al. 1996; USEPA 1994). Our findings of prolonged emergence time under Pd contamination (Figure S9) reinforce this concept. Conversely, although Pt caused rapid and complete mortality at low tested concentrations, it did not significantly affect the emergence time of surviving individuals, suggesting a more acute mode of action.

Regarding adult biomass and sexual dimorphism, the lack of significant effect of metal concentration on adult weight (Figure S10), along with the observed attenuation of the natural sexual dimorphism (females heavier than males) under Pd and Pt exposures, suggests that these metals may differentially affect female growth. This novel observation warrants further investigation, as it may reflect the disruption of sex‐specific growth or developmental mechanisms in *C. riparius* . Finally, the absence of significant effects of Pd or Pt on adult sex ratio indicates that this endpoint is a less sensitive one compared with survival or emergence time. Overall, these data support the view that growth and emergence time are more sensitive indicators of metal toxicity in sediments than survival alone, in line with multiple previous studies (Call et al. 1993; Taylor et al. 1993; USEPA 1994).

### 4.6 Ecotoxicological Implications

#### 4.6.1 Environmental Contents

Our study reveals toxicity thresholds for Pd and Pt that far exceed environmental concentrations (Table S5), as high exposure levels were necessary to elicit measurable effects and establish relevant toxicity reference values. When considering LOECs (Figure S9 and Table S8), values are between 37 and 804 μg·g^−1^ dw. Previous works indicate Pd and Pt concentrations in environmental sediments (Table S10) ranging from 0.3 to 90.8 ng·g^−1^ dw (0.0003–0.0908 μg·g^−1^ dw). Therefore, the average environmental concentration (~0.0455 μg g^–1^) is nearly 9.500 times lower than the average LOEC (~420.5 μg g^–1^) determined in our study. When considering acute LC_50_ values, which range from 52 to 1192 μg·g^−1^ dw, the average is approximately 622 μg·g^−1^ dw. This means that the average LC_50_ is approximately 13.500 times higher than the average environmental concentration in the literature. Yet it remains important to consider that hospital wastewaters represent a significant emerging source of high contents of Pt and Pd in the environment (Abdou et al. 2018; Kümmerer et al. 1999). For example, average daily concentrations of hospital effluent from five European hospitals were around 601 ng·L^−1^ Pt, with variations from 2 to 3580 ng·L in 2‐h mixed samples (Kümmerer et al. 1999). These findings highlight that Pt and Pd can reach elevated concentrations locally and intermittently in the water column. Supporting Information S7 explores ecotoxicological aspects such as analytical difficulties in measuring PGEs and the effects of organic matter on their behavior and toxicity, both of which are important to consider when evaluating previous findings and planning future investigations.

#### 4.6.2 Comparing the Toxicity of Other Metals

As shown in Table S11, the mean acute toxicity (LC_50_) values determined in our study—503 mg·kg^−1^ for Pd and 178 mg·kg^−1^ for Pt—position these PGEs among metals of moderate to high toxicity for freshwater benthic invertebrates. Although Pd and Pt appeared less acutely toxic than certain nonessential and classically recognized toxic metals such as cadmium (LC_50_ = 39 mg·kg^−1^) for *C. riparius* (Milani et al. 2003) and lead (LC_50_ ≈ 7.2 mg·kg^−1^) for *H. azteca* (Borgmann and Norwood 1999), their toxicity exceeded that of uranium (LC_50_ = 2442 mg·kg^−1^) for *H. azteca* (Liber et al. 2011), nickel (LC_50_ = 665 mg·kg^−1^) for *C. riparius* (Milani et al. 2003), arsenic (LC_50_ = 532 mg·kg^−1^) for *H. azteca* , and molybdenum (LC_50_ > 3742 mg·kg^−1^) for *H. azteca* (Liber et al. 2011). Importantly, the toxicity of Pd and Pt was in the same range as that of copper—a well‐known ecotoxic metal—for both *H. azteca* (128–316 mg·kg^−1^) and *C. riparius* (320–402 mg·kg^−1^) (Milani et al. 2003; Roman et al. 2007).

These considerations emphasize the toxic potential of Pd and Pt, elements still poorly represented in regulatory frameworks. Given their increasing use and environmental release, these findings support the urgent need to further investigate their environmental fate and bioavailability, as well as short‐ and long‐term effects on aquatic ecosystems. In this context, Pd and Pt must be considered as emerging contaminants of concern, deserving greater attention in both incoming monitoring programs and ecological risk assessments.

#### 4.6.3 Contributions and Limitations of the Study

In our testing conditions, we use 50% natural sediment and 50% quartz sand mixture, resulting in a low organic carbon content (~0.5%; Table S1). This choice better mimics natural conditions by providing organic matter directly from field sediments rather than the artificial sources (e.g., α‐cellulose) commonly used in sediment tests (Carney Almroth et al. 2023; Lacey et al. 1999). This approach also aimed to limit the influence of organic carbon on the bioavailability and toxicity of Pd and Pt, as high organic matter content can complex metals and reduce their toxicity (Boukhari et al. 2025). Such low organic carbon levels are similar to the values reported in certain clay‐rich natural environments, such as Lake Saint‐Pierre near Grondines (Environnement Canada and MDEPQ 2007) and the Chaudière River, from which our sediments were collected. In addition, as we added food during the exposure experiments, the organic carbon content is expected to increase over time, making our experimental design more realistic. Nonetheless, we acknowledge that the use of mixed sediments may limit direct extrapolation to all freshwater systems and recommend further studies across a wider range of sediment and organic carbon compositions.

Here, we did not measure either metal in porewater/overlying water nor acid volatile sulfide (AVS) in tested sediments, but it is highly recommended to do such analyses in future works. As highlighted by Liber et al. (2011), data on porewater metal concentrations in toxic sediments would be a useful addition to future guideline documents. Future work should, therefore, consider them to better characterize exposure pathways and refine toxicity interpretation for both benthic and epibenthic organisms. Despite such limitations mentioned before, the experimental design used in the present work to assess Pd and Pt toxicity covers a real knowledge gap regarding the toxicity of both metals, since such data are not available in the literature yet.

## 5 Conclusion

By exposing two freshwater benthic invertebrates to sediments contaminated with Pd or Pt, this study provides novel insights into the ecotoxicological risks associated with these emergent metals. Results reveal clear species‐, exposure time‐, and endpoint‐specific responses. As initially hypothesized, Pt exhibited greater acute toxicity, particularly for survival, whereas Pd exerted stronger effects on sublethal endpoints such as growth and development. The benthic sediment‐dwelling *Chironomus riparius* accumulated both metals to a greater extent than the epibenthic *H. azteca* , likely due to differences in their feeding behavior. *H. azteca* bioaccumulated more Pt, whereas *C. riparius* accumulated more Pd. Chronic exposure experiments further indicated that Pd induced more pronounced effects than Pt on survival and delayed emergence time in *C. riparius* . Although no significant alteration of sex ratio was observed, a significant reduction in adult female weight was noted, pointing to natural dimorphism alteration associated with Pd exposure.

Importantly, although current environmental concentrations remain below the observed effect thresholds, the rapid expansion in industrial use of PGEs raises legitimate concerns regarding their long‐term accumulation and ecological impact. Moreover, findings confirm that Pd and Pt present moderate to high toxicity, comparable with other nonessential metals. This study lays the groundwork for advancing sediment ecotoxicology and highlights the pressing need to integrate PGEs into sediment quality criteria. Further investigations should focus on Pd–Pt mixtures and their modes of action (using omic approaches, subcellular metal partitioning approach) to enhance risk assessment strategies and improve predictive models for freshwater ecosystem protection.

## Author Contributions

**Alice Carle:** conceptualization, methodology, validation, formal analysis, writing – original draft, writing – review and editing, visualization. **Ludivine Preizal:** methodology, writing. **Marc Amyot:** conceptualization, supervision, project administration, funding acquisition. **Maikel Rosabal:** conceptualization, supervision, project administration, funding acquisition.

## Conflicts of Interest

The authors declare no conflicts of interest.

## Supporting information


**Figure S1:** Experimental timeline for acute toxicity tests on *Hyalella azteca* (A) and *Chironomus riparius* (B), and chronic tests on *C. riparius* (C), with Pt (blue diamonds) and Pd (black circles). Negative days indicate the sediment equilibration period required to reach pH conditions suitable for organism survival.
**Figure S2:** Survival (%) as a function of the measured content (μg_metal_·g_sed_^−1^ dw) of Pd (black points) and Pt (blue rhombuses) following exposure of *H. azteca* (H) and *C. riparius* (C). Toxicological parameters (NOEC and LOEC = no‐observed‐ and lowest‐observed‐effect concentration) are shown. Asterisks (*) denote significant differences compared with control condition (*p* ≤ 0.05, *n* = 4).
**Figure S3:** Linear regression curves for relative growth rate (%) as a function of the measured content (μg_metal_·g_sed_^−1^ dw) of Pd (black points) and Pt (blue rhombuses) following exposure of *H. azteca* (H) and *C. riparius* (C). Adjusted *R*‐squared and *p*‐values estimated are shown.
**Figure S4:** Linear regression curves for relative growth rate (%) as a function of the survival (%) of Pd (black points) and Pt (blue rhombuses) following exposure of *H. azteca* and *C. riparius* , respectively. Adjusted *R*‐squared and *p*‐value estimated are shown.
**Figure S5:** Linear regression curves for bioaccumulation (μg_metal_·g_organism_^−1^ dw) as a function of the measured concentration (μg_metal_·g_sed_^−1^ dw) of Pd (black points) and Pt (blue rhombuses) following exposure of *H. azteca* (H) and *C. riparius* (C). Adjusted *R*‐squared and *p*‐values estimated are shown.
**Figure S6:** Linear regression curves for survival (%) and for relative growth rate (%) as a function of the bioaccumulation (μg_metal_·g_organism_^−1^ dw) of Pd (black points) and Pt (blue rhombuses) following exposure of *H. azteca* (H) and *C. riparius* (C). Adjusted *R*‐squared and *p*‐values estimated are shown.
**Figure S7:** Survival (%) as a function of the measured content (μg_metal_·g_sed_^−1^ dw) of Pd (black points) and Pt (blue rhombuses) following exposure of *C. riparius* . Toxicological parameters (NOEC and LOEC = no‐observed‐ and lowest‐observed‐effect concentration) are shown in the table below. Asterisks (*) denote significant differences compared with control condition (*p* ≤ 0.05, *n* = 4).
**Figure S8:** Sex ratio (%) as a function of the measured content (μg_metal_·g_sed_^−1^ dw) of the emerged *C. riparius* for Pd (black points) and Pt (blue rhombuses). The cross symbol (✚) denotes the absence of data at this content due to complete mortality of the exposed organisms. Any significant difference compared with control conditions after a Kruskal–Wallis test followed by Dunn's test with Bonferroni adjustment was found.
**Figure S9:** Emergence (days) as a function of the measured content (μg_metal_·g_sed_^−1^ dw) of the emerged *C. riparius* for Pd (black points) and Pt (blue rhombuses). Toxicological parameters (NOEC; LOEC) are shown in the table below. The cross symbol (✚) denotes the absence of data at this content due to complete mortality of the exposed organisms. Asterisks (*) denote significant differences compared with control condition (*p* ≤ 0.05, *n* = 4). ND: not determined.
**Figure S10:** Flying adult weight (mg) as a function of the measured concentration (μg_metal_·g_sed_^−1^) of the emerged *C. riparius* for Pd (black points) and Pt (blue rhombuses). The cross symbol (✚) denotes the absence of data at this content due to complete mortality of the exposed organisms. A Kruskal–Wallis test denoted no significant impact of metal content on adult weight. A Wilcoxon–Mann–Whitney test showed significant differences between female and male weight for conditions shown by an asterisk (*).
**Table S1:** Characterization of trace element composition and various parameters in tested sediments (50% of natural sediment from the Chaudière River (Quebec, Canada); 50% Carib Sea Super Natural Moonlight Sand artificial sediment).
**Table S2:** Values (mean ± SD; *n* = 28 for the controls and *n* = 81 for the conditions) of the physicochemical parameters (pH, temperature, dissolved oxygen, conductivity, nitrites, nitrates, and ammonium levels) in the water during the beginning, middle, and end of acute and chronic tests.
**Table S3:** Recovery percentages (mean ± standard deviation) of platinum and palladium in the certified reference materials (CRMs), and the spiked materials after ICP‐QQQ analysis (e.g., matrice or CRMs [+ spiked quantity, ng·g^−1^]).
**Table S4:** Metal content (mean ± standard deviation, μg_metal_·g_sed_^−1^) in the metal‐contaminated sediments at the beginning and the end of exposures for each test compared with the nominal content.
**Table S5:** Toxicological parameters derived from concentration–response curves for *H. azteca* and *C. riparius* exposed to Pd‐ and Pt‐contaminated sediments during 10 and 14 days, respectively.
**Table S6:** Summary of toxicological parameters for relative growth rate (%) in *H. azteca* and *C. riparius* exposed to measured concentrations of Pd and Pt in sediments.
**Table S7:** Summary of toxicological parameters for bioaccumulation (μg_metal_·g_organism_^−1^ dw) in *H. azteca* and *C. riparius* exposed to measured concentrations of Pd and Pt in sediments.
**Table S8:** Toxicological parameters derived from concentration–response curves for *C. riparius* exposed to Pd‐ and Pt‐contaminated sediments during 28 days.
**Table S9:** Summary of median lethal concentration (LC_50_) and median effective concentration (EC_50_) values reported in the literature for the toxicity of soil and water contaminated with palladium or platinum metals (Pd: palladium; Pt: platinum) on terrestrial and aquatic invertebrate species. Our values are reported at the end of the table.
**Table S10:** Summary of palladium and platinum accumulation in several environmental compartments (sediment, soil, road dust, surface water) in environments located around operating mines (Table A) and along roads in several countries (Table B).
**Table S11:** Summary of median lethal concentration (LC_50_) and median effective concentration (EC_50_) values reported in the literature for toxicity of sediment contact test contaminated with class‐B and borderline metals (Cu: copper; Ni: nickel; U: Uranium; Hg: mercury; Pb: lead; Zn: zinc; Cd: cadmium; As: arsenic; Ag: silver; Mo: Molybdène) on *C. riparius* or *dilitus* and *Hyaella azteca* species.

## Data Availability

The data that support the findings of this study are available from the corresponding author upon reasonable request.
