# Supplementary material for: Acute and Chronic Toxicity of Sediments Containing Platinum and Palladium on Freshwater Benthic Organisms Chironomus riparius and Hyalella azteca
Source: J Appl Toxicol. 2025 Oct 2;46(4):1151–63. doi: 10.1002/jat.4933 (PMC12945464; doi:10.1002/jat.4933)
Supplement: Supplementary file 1 — Figure S1: Experimental timeline for acute toxicity tests on Hyalella azteca (A) and Chironomus riparius (B), and chronic tests on C. riparius (C), with Pt (blue diamonds) and Pd (black circles). Negative days indicate the sediment equilibration period required to reach pH conditions suitable for organism survival. Figure S2: Survival (%) as a function of the measured content (μgmetal·gsed −1 dw) of Pd (black points) and Pt (blue rhombuses) following exposure of H. azteca (H) and C. riparius (C). Toxicological parameters (NOEC and LOEC = no‐observed‐ and lowest‐observed‐effect concentration) are shown. Asterisks (*) denote significant differences compared with control condition (p ≤ 0.05, n = 4). Figure S3: Linear regression curves for relative growth rate (%) as a function of the measured content (μgmetal·gsed −1 dw) of Pd (black points) and Pt (blue rhombuses) following exposure of H. azteca (H) and C. riparius (C). Adjusted R‐squared and p‐values estimated are shown. Figure S4: Linear regression curves for relative growth rate (%) as a function of the survival (%) of Pd (black points) and Pt (blue rhombuses) following exposure of H. azteca and C. riparius , respectively. Adjusted R‐squared and p‐value estimated are shown. Figure S5: Linear regression curves for bioaccumulation (μgmetal·gorganism −1 dw) as a function of the measured concentration (μgmetal·gsed −1 dw) of Pd (black points) and Pt (blue rhombuses) following exposure of H. azteca (H) and C. riparius (C). Adjusted R‐squared and p‐values estimated are shown. Figure S6: Linear regression curves for survival (%) and for relative growth rate (%) as a function of the bioaccumulation (μgmetal·gorganism −1 dw) of Pd (black points) and Pt (blue rhombuses) following exposure of H. azteca (H) and C. riparius (C). Adjusted R‐squared and p‐values estimated are shown. Figure S7: Survival (%) as a function of the measured content (μgmetal·gsed −1 dw) of Pd (black points) and Pt (blue rhombuses) following exposure of [file JAT-46-1151-s001.docx]

**Supporting Information**

**Acute and chronic toxicity of sediments containing platinum and palladium on two freshwater benthic organisms *Chironomus riparius* and *Hyalella azteca***

Alice Carle^1^, Ludivine Preizal^1^, Marc Amyot^2^, Maikel Rosabal^1^

^1^ Département des sciences biologiques, Laboratoire d'Analyses Environnementales, Université du Québec à Montréal, 141, Avenue du Président-Kennedy, Montréal, Québec, H2X 1Y4, Canada.

^2^ Département de sciences biologiques, Complexe des sciences, Université de Montréal, 375 Avenue Thérèse-Lavoie-Roux, Montréal (Québec), H2V 0B3

**Materials and Methods**

**Supplemental information 1. Sediment contamination**

After one night at temperature room (16°C), contaminated sediment was homogenized again and 100 g (acute-) or 150 g (chronic-tests) was introduced in each of six individual exposure jars (replicates). Then, 350 mL (acute) or 400 mL (chronic) of reconstituted water was added, and each jar was placed in the environmental chambers under continuous aeration. To ensure good homogenization and pH stability above 7 in all exposure jars, an equilibration period of 7-days was applied, including three water changes every 2-days for Pd. For Pt, an equilibration period of 3-days for Pt one water change after 2-days was applied.

**Supplemental information 2. Biological parameters measurements**

*Acute exposure*: once the 14- and the 10-days exposures completed for *Hyalella azteca* and *Chironomus riparius* respectively, survival and growth were estimated. Surviving organisms were handpicked, photographed using AmLite (Amscope) software connected to a camera (Amscope; MU1803 model). Growth inhibition was assessed by measuring the body length using ImageJ software.

*Chronic exposure*: the survival, the time to emergence, the sex ratio of emerged adults as well as the weight of flying ones were considered as toxicity endpoints for the 28-days exposures of *C. riparius*. Emerged adults were captured every day during the emergence period using a metal-free tube (VWR) and the weight of each individual was recorded discriminating flying and non-flying adults, as well as males and females for further analysis (Nieto et al., 2017; Saraiva et al., 2020). Adult molts from the metamorphosis of larvae into flying adults were collected at each water changes every five days and rinsed using the same method as for the larvae. Adults and molts were stored at -20°C for future analysis.

**Supplemental information 3. Quality control of toxicity tests**

All toxicity tests met the Test Acceptance Criteria as described in the OECD guideline 218 (*Sediment-Water Chironomid Toxicity Using Spiked Sediment*, OECD, 2004) for *Chironomus riparius*, and ISO 16303 (*Water quality — Determination of toxicity of freshwater sediments using* *Hyalella azteca*, ISO, 2013) as well as Canadian methods for culture maintenance (CEAEQ DR-09-BMS-027), sediment preparation (Environnement Canada, 1995), organism exposures (Environnement Canada, 1997; 2017), preparation, and biological testing of sediments. A control using KCl was conducted to check the influence of the Cl contained in the commercial metal salts. The Cl content of the solution was adjusted to reach the maximum content of Cl from PGE. A positive control of mortality using CuSO_4_ (Anachemia Canada Inc), at a concentration of 3 mg.L^−1^ for which 100% mortality is expected, was conducted to check the sensitivity of the testing organisms (Cairns et al., 1984; Environnement Canada, 1995).

First, the metal content and physicochemical characteristics of the sediments, as well as those of water (pH, temperature, dissolved oxygen, conductivity, ammonium, nitrite, and nitrate levels) monitored throughout the experiment, have proven to be compatible with the proper development of *Chironomus riparius* and *Hyalella azteca* studied here (Tables SI.II; III).

Then, more than 80% survival was observed in both controls – the chloride control and the negative control without metal – while no survival was observed in the positive control made of copper. Given that all exposure jars were handled in the same manner, this suggests that organisms were affected by the presence of metals within the sediments and not by the chloride had with the metal salts, and that culture organisms were originally healthy and sensitive. Since that no significant differences were detected between the negative (n=4) and the chloride (n=4) controls, both conditions have been compiled into a single control group (n=8) for data analysis.

**Supplemental information 4. Organism digestion procedure**

Then, 600 μl of metal-trace HNO_3_ (Fisher Scientific), purified by sub-wetting with the DST-1000 purification system (Savillex), and 200 μl of ultra-trace HCl (Fisher Scientific) were added, and was left overnight. Samples were then digested at 120°C in a digestion block for 3-h using an electric sterilizer (All American, model 50X-120V). After cooling, 250 μl of ultra-trace hydrogen peroxide (Fisher Scientific; 30% H_2_O_2_ Optima grade) were added to ensure the complete removal of organic matter. The digestate was placed in a fume hood for 24-h at room temperature, transferred to 15 mL trace metal-free tubes (VWR), and diluted with Milli-Q water to achieve a final volume of 15 mL corresponding to a final acid concentration of 1% HNO_3_ and 5% HCl (v/v). Metals internalized in biological fractions were measured using an inductively coupled plasma-mass spectrometer triple quadrupole (Agilent, ICP-MS/MS, 8990) at *Université de Montréal* (UdeM).

**Supplemental information 5. Quality control of measured Pd and Pt contents by ICP-MS/MS**

Certified reference materials (CRM) were used to ensure the highest accuracy and reliability of the measurements, and digestion analytical blanks were undergone to control contamination. Quality controls follow the same steps in the analytical protocol as the samples. For metal contained in sediments, measurement quality for Pd and Pt was tested using control sediment spiked using 200 µL of 99.9 µg.mL^-1^ solution of Pd and Pt ICP standards (999 ± 4 ug.ml^-1^, SCP SCIENCE). For metal internalized in organisms, accuracy and precision of Pt measurements were evaluated using the CRM IAEA-450 (International Atomic Energy Agency; unicellular microalga *Scenedesmus obliquus*). The IAEA-450 spiked with 0.1 and 0.01 µg.L^-1^ of Pd ICP standard were used to evaluate the quality of Pd measurements. Metal spiking contents were selected to ensure that, after dilution, the resulting levels fell within the standard curve of the ICP-MS for these elements. Results of those qualitative analyses and recovery percentages are shown in the Table SI.III. In addition, the relation between nominal metal contents and measured contents (in %) for both metals in each toxicity test is given in Table SI.IV.

**Discussion**

**Supplemental information 6. Factors influencing metal uptake in aquatic organisms**

It is important to note that bioaccumulation data on various aquatic invertebrates indicates that the uptake mechanism of metals depends on several environmental factors. As an example, observed in the isopod *Asellus aquaticus*, the pH influenced the bioaccumulation of Pt (Rauch & Morrison, 1999). Exposure time also influenced metal uptake by organisms. Indeed, as observed in the zebra mussel *Dreissena polymorpha* (Singer et al., 2005) and the freshwater mussel *Elliptio complanata* (Mays, 2009), uptake kinetics revealed that Pt levels remained stable in organisms throughout the exposure period, whereas Pd levels kept increasing. The mobility of the PGE is also a key factor of their bioaccumulation. Due to its predominant neutral charge, Pd exhibits higher mobility than other PGE (Leopold et al., 2017), while Pt mobility can be enhanced by the presence of high chloride concentration or by low pH (Zereini et al., 1997). Other factors such as the presence of organic matter and humic acids play an important role. This explained how the bioaccumulation of Pt in the green alga *Chlamydomonas reinhardtii* is greater in the presence of natural organic matter (Hourtané et al., 2022; Rioux, 2018). Similarly, the presence of humic acids favors the bioaccumulation of Pt and Pd in the zebra mussel (Zimmermann et al., 2005).

An important factor influencing metal bioavailability and toxicity in sediments that was not explicitly measured in this study is the concentration of acid volatile sulfides. Those compounds can bind with metals such as Pd and Pt, reducing their bioavailability by forming insoluble metal-sulfide complexes. The presence and concentration of acid volatile sulfides in sediments can therefore significantly modulate metal toxicity on organisms as reported by Di Toro et al. (1992) for cadmium toxicity. Future studies should include AVS measurements to better understand metal partitioning and bioavailability dynamics in similar sediment toxicity assessments.

**Supplemental information 7. PGE measurement issues and organic matter influence on their behavior**

Although, it is crucial to note that adequate measuring of PGE in the environment is challenging because of inexistent certified reference material for Pd, trace contents and metal interferences (Bluteau et al., 2025; Obata et al., 2006). Accurate analysis of PGE contents in water, sediment, or biological samples necessitate rigorous analytical procedures, including pre-treatment and pre-concentration steps tailored to the specific matrix being analyzed (Obata et al., 2006; Schindl & Leopold, 2015).

Recent findings of Bluteau et al. (2025) confirmed rhodium (Rh), Pd, and Pt concentration gradients from road dust to sediments, soils, and water, indicating their progressive dispersion through the environment. However, the findings differ from earlier studies concerning biological contamination, showing low PGE detection in wild organisms and highlighting the need for caution in interpreting elevated PGE reports. Further research is needed to clarify PGE bioaccumulation dynamics and improve the reliability of contamination assessments.

Nevertheless, with projected increases in emissions of these metals, their gradual accumulation in waterways and coastal sediments could eventually lead to future metal concentrations reaching toxic thresholds. It is therefore essential to define clear toxicological values for Pd and Pt to define releasing limits for Pd and Pt in industrial effluents, particularly from the automotive, mining, electronics, and medical sectors.

Future studies measuring PGE should incorporate these considerations to ensure the reliability of the results. The metal analysis and recovery methods used in this study were optimized over several months, and recovery efficiencies – presented in Table S.III – consistently exceeded 90%.

Moreover, organic matter can modify the behavior and the effects of the PGEs (Hourtané et al., 2022, 2024; Rioux, 2018). Indeed, ionized forms of PGE may form complexes with organic matter which impact their mobility and bioavailability (Gerhardt, 1993; Lustig et al., 1998). For example, humic compounds in the soil have been shown to reduce the bioavailability and bioaccumulation of Pd in the zebra mussel *Dreissena polymorpha* (Sures & Zimmermann, 2007). In the algae *Chlorella fusca* and *Chlamydomonas reinhardtii*, an opposite effect of organic matter on bioavailability was observed for Pt (Hourtané et al., 2022, 2024), that is contrary to the biotic ligand model according to organic matter should reduce the bioavailability of metals (Paquin et al., 2002). Therefore, since such an interaction can affect the toxicity metals depending on the organism exposed, it is important to quantify the organic matter fraction for accurate environmental risk assessment and quality standard setting. While in our study, we did not directly measure the organic matter within sediment, its potential influence has been minimized by using a mixture of artificial sediment without organic matter, and natural sediment with natural organic matter in equal quantity. Moreover, all tests were performed with sediments from the same batch to ensure similar content of organic matter between experiments.


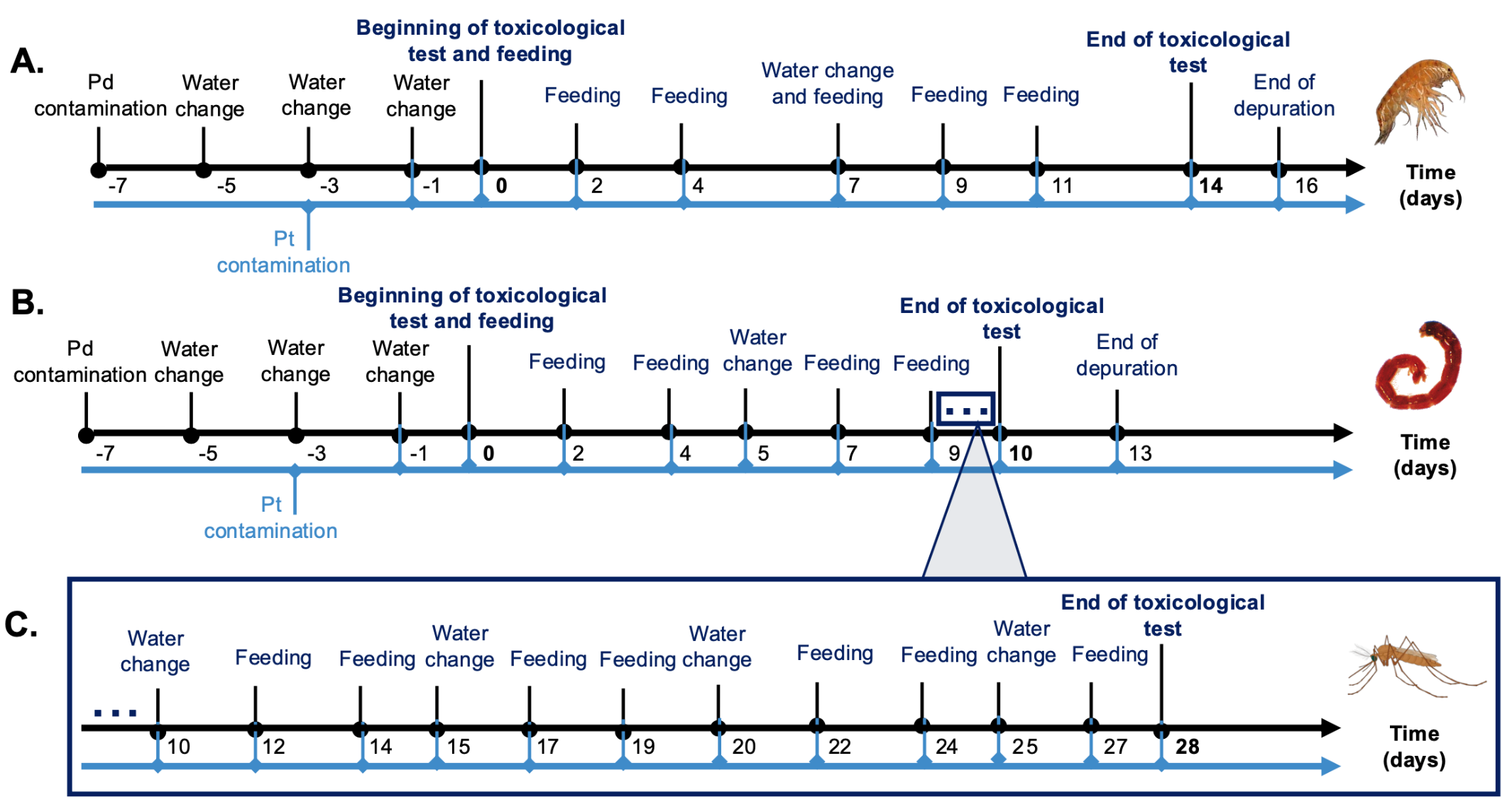


**Figure SI.1** Experimental timeline for acute toxicity tests on *Hyalella azteca* (A) and *Chironomus riparius* (B), and chronic tests on *C. riparius* (C), with Pt (blue diamonds) and Pd (black circles). Negative days indicate the sediment equilibration period required to reach pH conditions suitable for organism survival.

**Pd-H Pt- H**


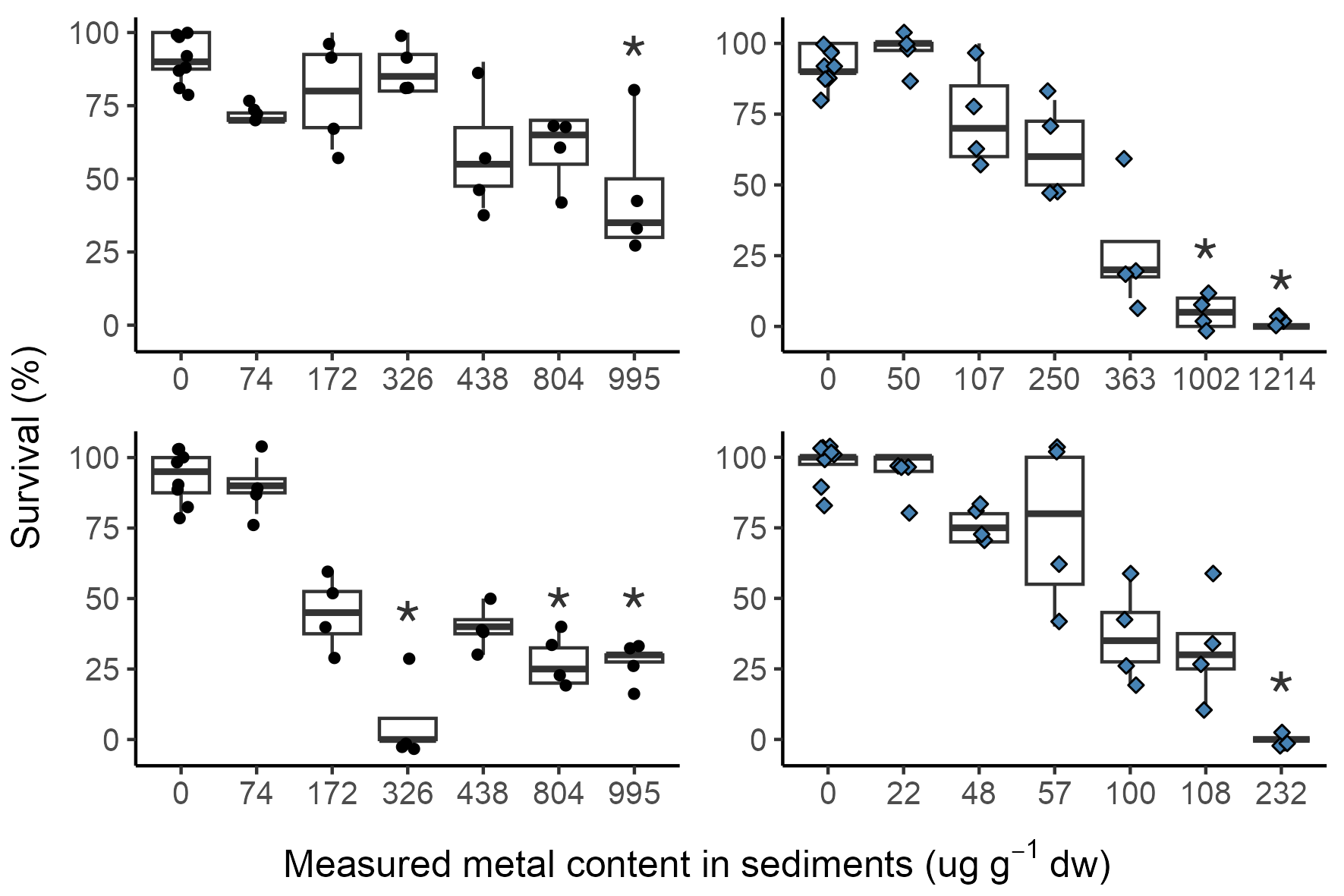


**Pd-C Pt- C**

| Organism | Parameter | Concentration (ug.g^-1^dw) | |
| --- | --- | --- | --- |
|  |  | Pd | Pt |
| *Hylella azteca* | NOEC | 804 | 1002 |
|  | LOEC | 995 | 1214 |
| *Chironomus riparius* | NOEC | 172 | 108 |
|  | LOEC | 326 | 232 |

**Figure SI.2** Survival (%) as a function of the measured content (μg_metal_.g_sed_-^1^ dw) of Pd (black points) and Pt (blue rhombuses) following exposure of *H. azteca* (H) *and C. riparius* (C). Toxicological parameters (NOEC and LOEC = no-observed- and lowest-observed-effect concentration) are shown. Asterisks (*) denote significant differences compared to control condition (p ≤ 0.05, n=4).


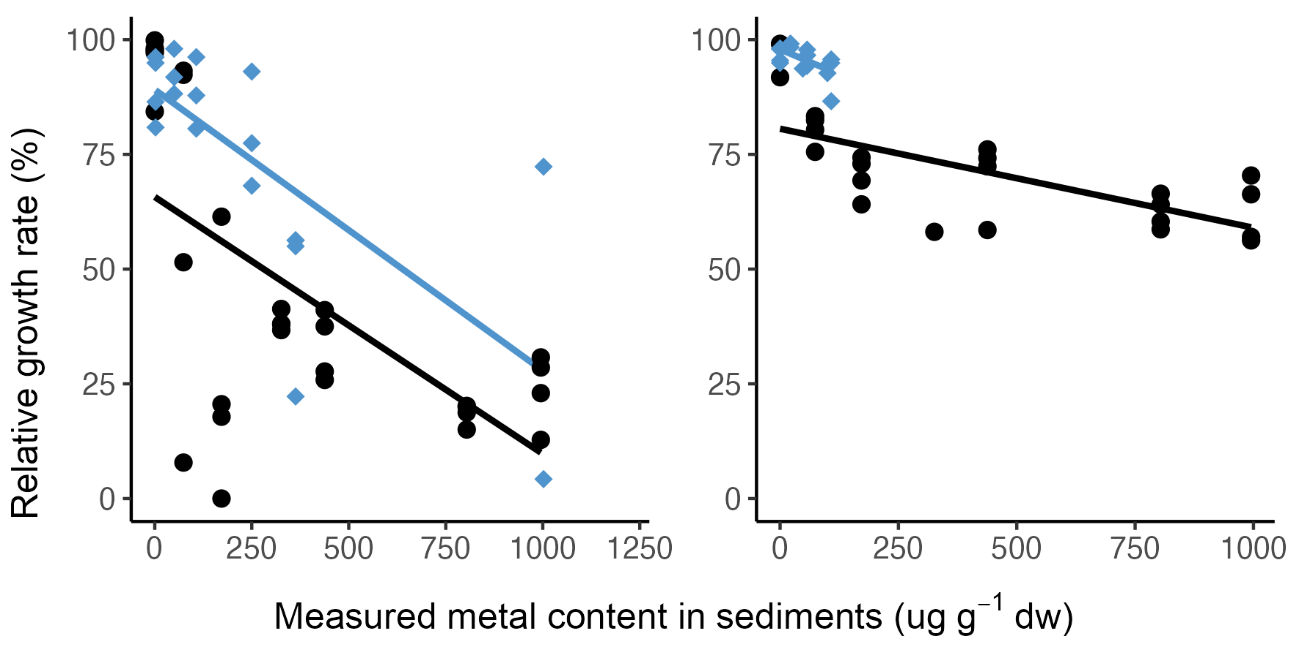


R^2^ = 0.50

p < 0.001

R^2^ = 0.10

p = 0.04

R^2^ = 0.60

p < 0.001

R^2^ = 0.40

p < 0.001

**H C**

**Figure SI.3** Linear regression curves for relative growth rate (%) as a function of the measured content (μg_metal_.g_sed_-^1^ dw) of Pd (black points) and Pt (blue rhombuses) following exposure of *H. azteca* (H) and *C. riparius* (C). Adjusted R-squared and p-values estimated are shown.


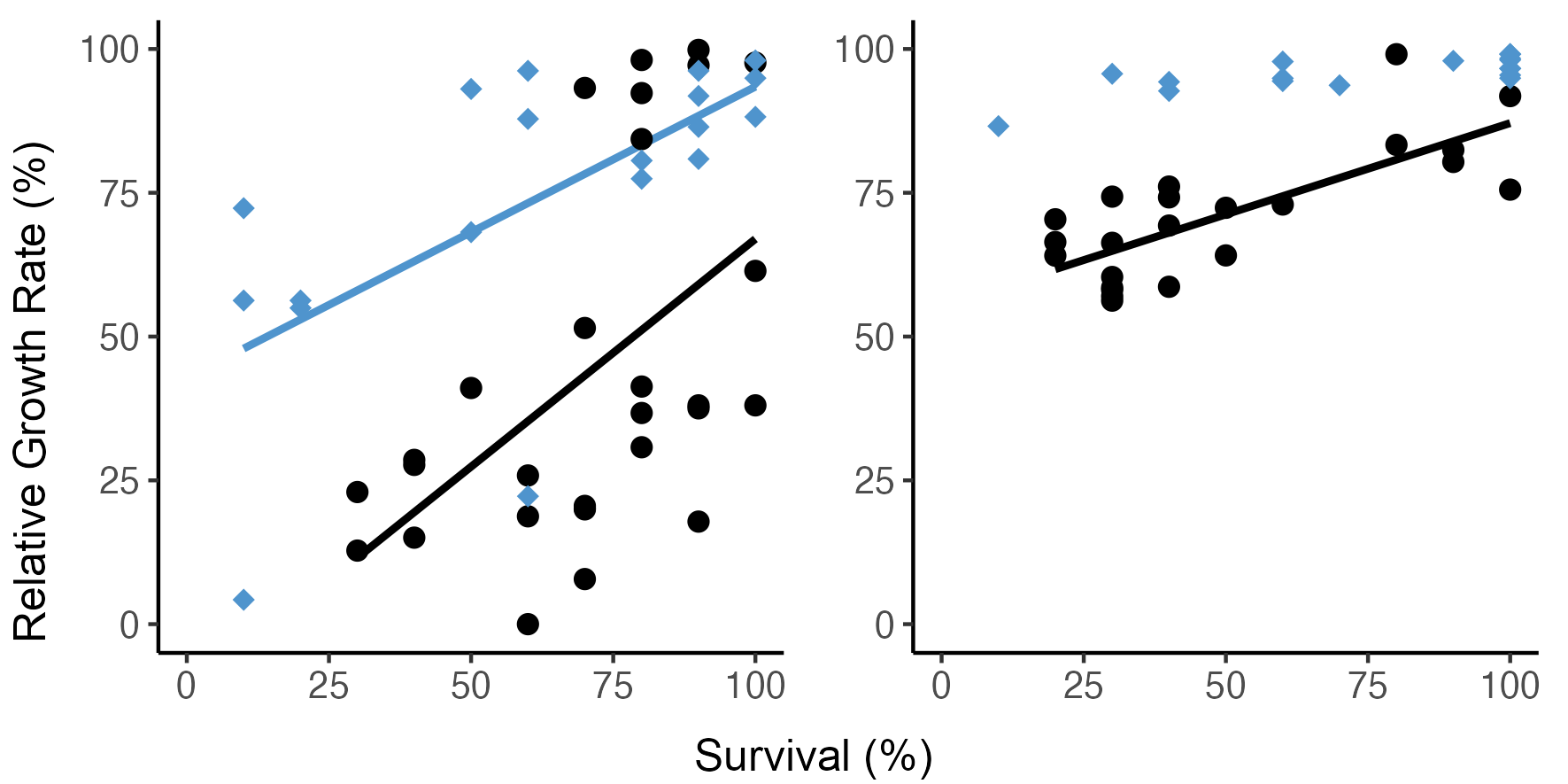


R^2^ = 0.70

p < 0.001

R^2^ = 0.35

p < 0.001

R^2^ = 0.50

p < 0.001

**H C**

**Figure SI.4** Linear regression curves for relative growth rate (%) as a function of the survival (%) of Pd (black points) and Pt (blue rhombuses) following exposure of *H. azteca* and *C. riparius*, respectively. Adjusted R-squared and p-value estimated are shown.

**
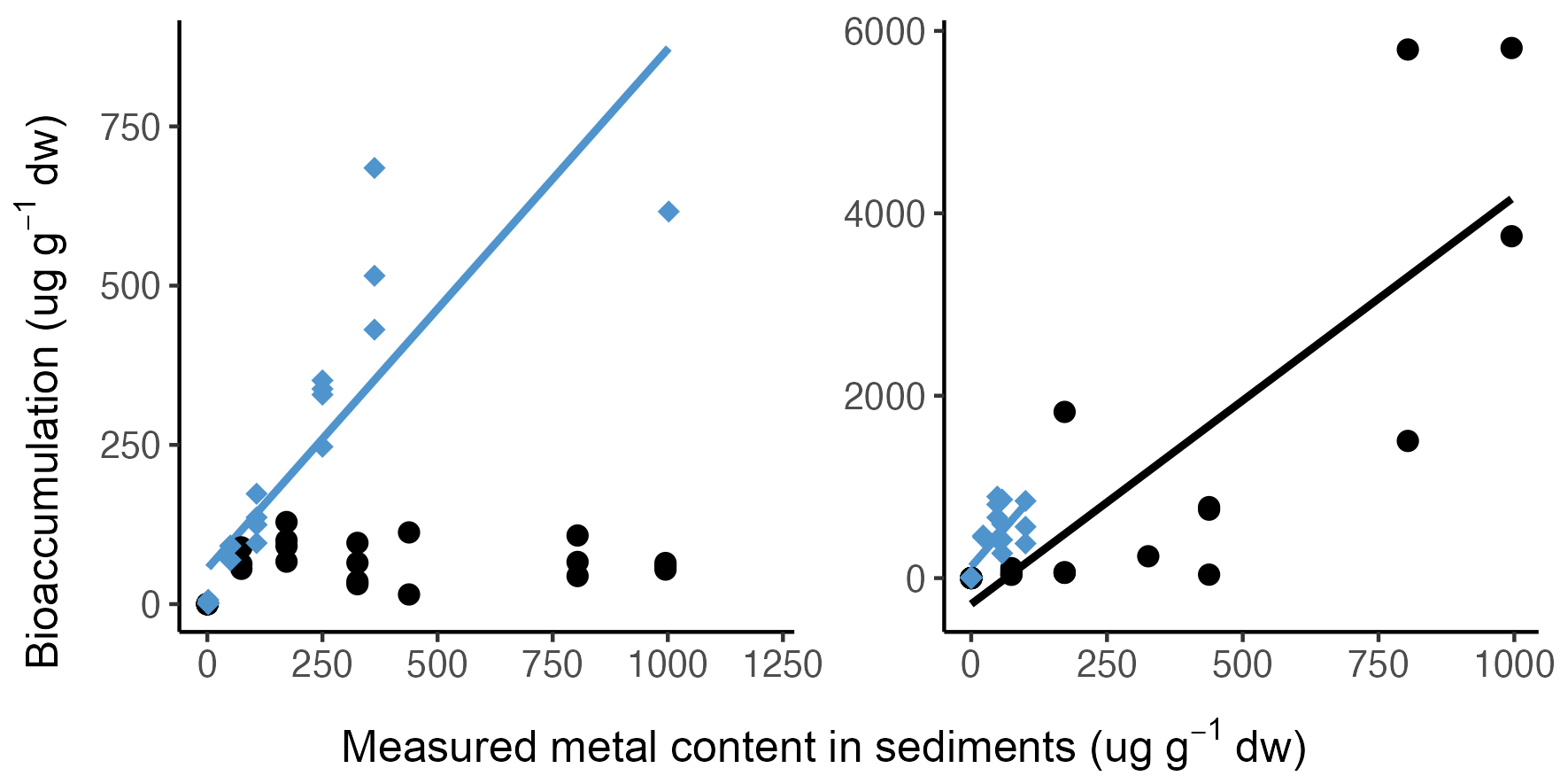
**

R^2^ = 0.71

p < 0.001

R^2^ = 0.50

p < 0.001

R^2^ = 0.74

p < 0.001

R^2^ = 0.14

p = 0.05

**H C**

**Figure SI.5** Linear regression curves for bioaccumulation (μg_metal_.g_organism_-^1^ dw) as a function of the measured concentration (μg_metal_.g_sed_-^1^ dw) of Pd (black points) and Pt (blue rhombuses) following exposure of *H. azteca* (H) and *C. riparius* (C). Adjusted R-squared and p-values estimated are shown.

R^2^ = 0.30

p = 0.008


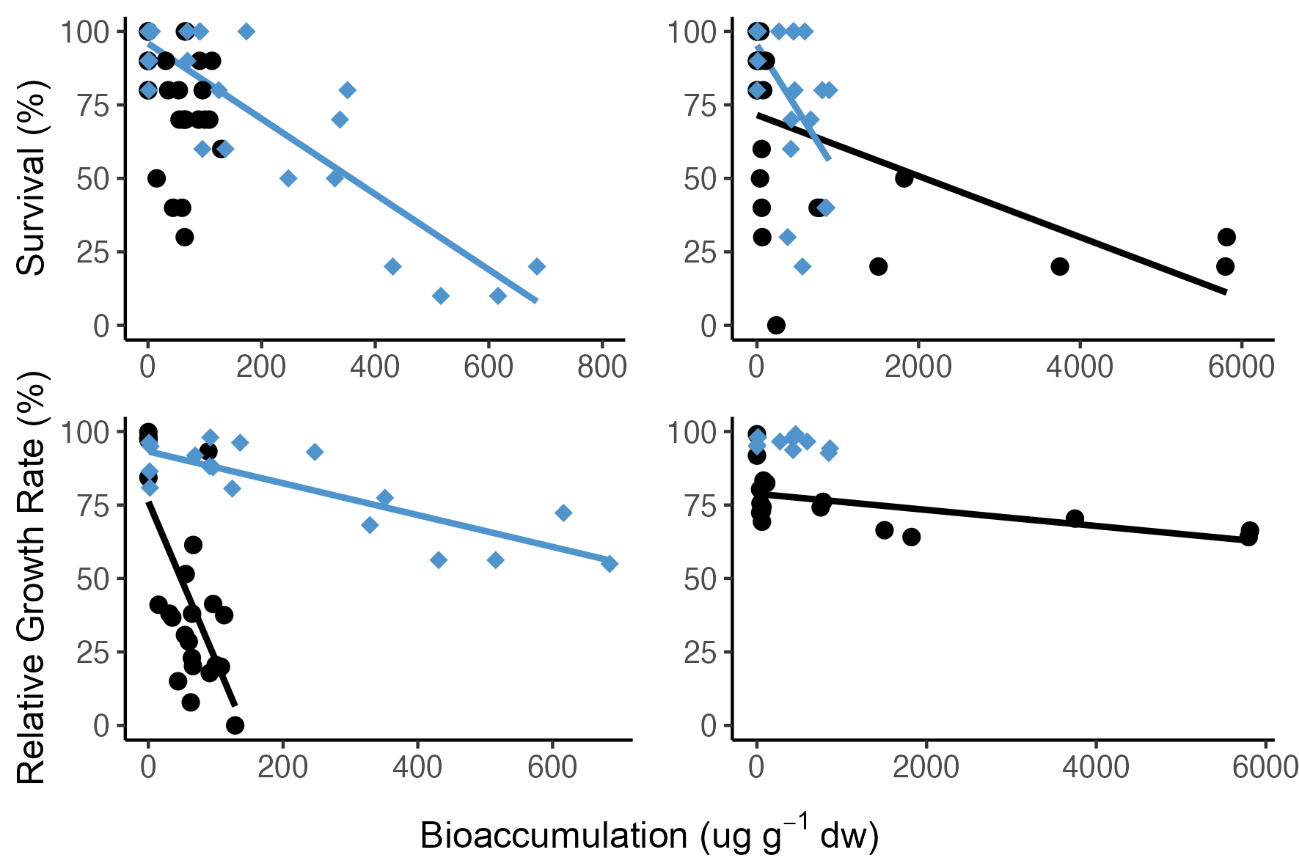


R^2^ = 0.50

p < 0.001

R^2^ = 0.50

p < 0.001

R^2^ = 0.04

p = 0.3

R^2^ = 0.30

p < 0.001

R^2^ = 0.30

p = 0.005

R^2^ = 0.80

p < 0.001

**H C**

**H C**

**Figure SI.6** Linear regression curves for survival (%) and for relative growth rate (%) as a function of the bioaccumulation (μg_metal_.g_organism_-^1^ dw) of Pd (black points) and Pt (blue rhombuses) following exposure of *H. azteca* (H) and *C. riparius* (C). Adjusted R-squared and p-values estimated are shown.


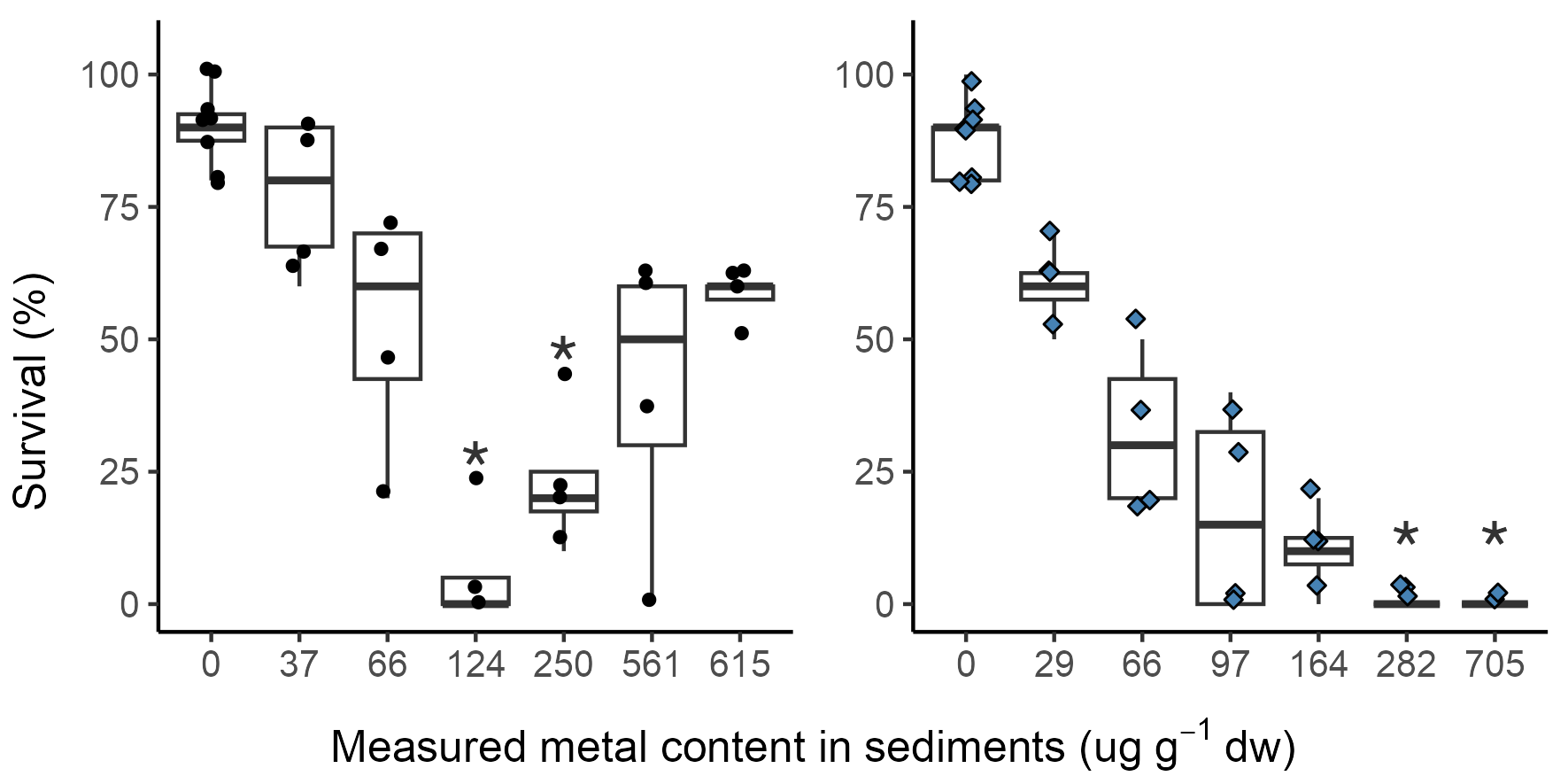


**Pd Pt**

| Parameter | Concentration ± (ug.g^-1^dw) | |
| --- | --- | --- |
|  | Pd | Pt |
| NOEC | 66 | 282 |
| LOEC | 124 | 164 |

**Figure SI.7** Survival (%) as a function of the measured content (μg_metal_.g_sed_-^1^ dw) of Pd (black points) and Pt (blue rhombuses) following exposure of *C. riparius*. Toxicological parameters (NOEC and LOEC = no-observed- and lowest-observed-effect concentration) are shown in the table below. Asterisks (*) denote significant differences compared to control condition (p ≤ 0.05, n=4).


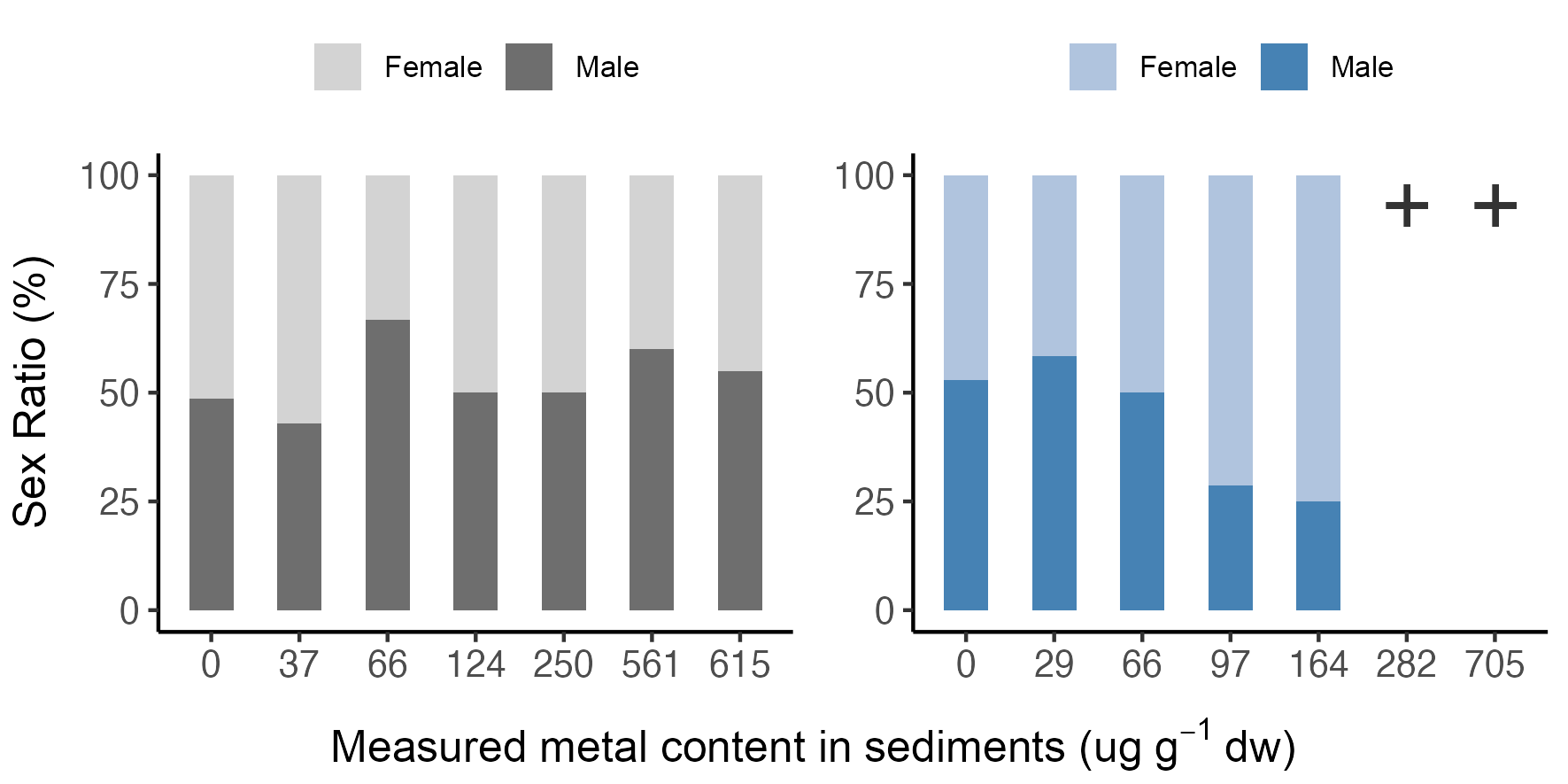


**Pd Pt**

**Figure SI.8** Sex ratio (%) as a function of the measured content (μg_metal_.g_sed_-^1^ dw) of the emerged *C. riparius* for Pd (black points) and Pt (blue rhombuses). The cross symbol (✚) denotes the absence of data at this content due to complete mortality of the exposed organisms. Any significant difference compared to control conditions after a Kruskal-Wallis test followed by Dunn's test with Bonferroni adjustment was found.


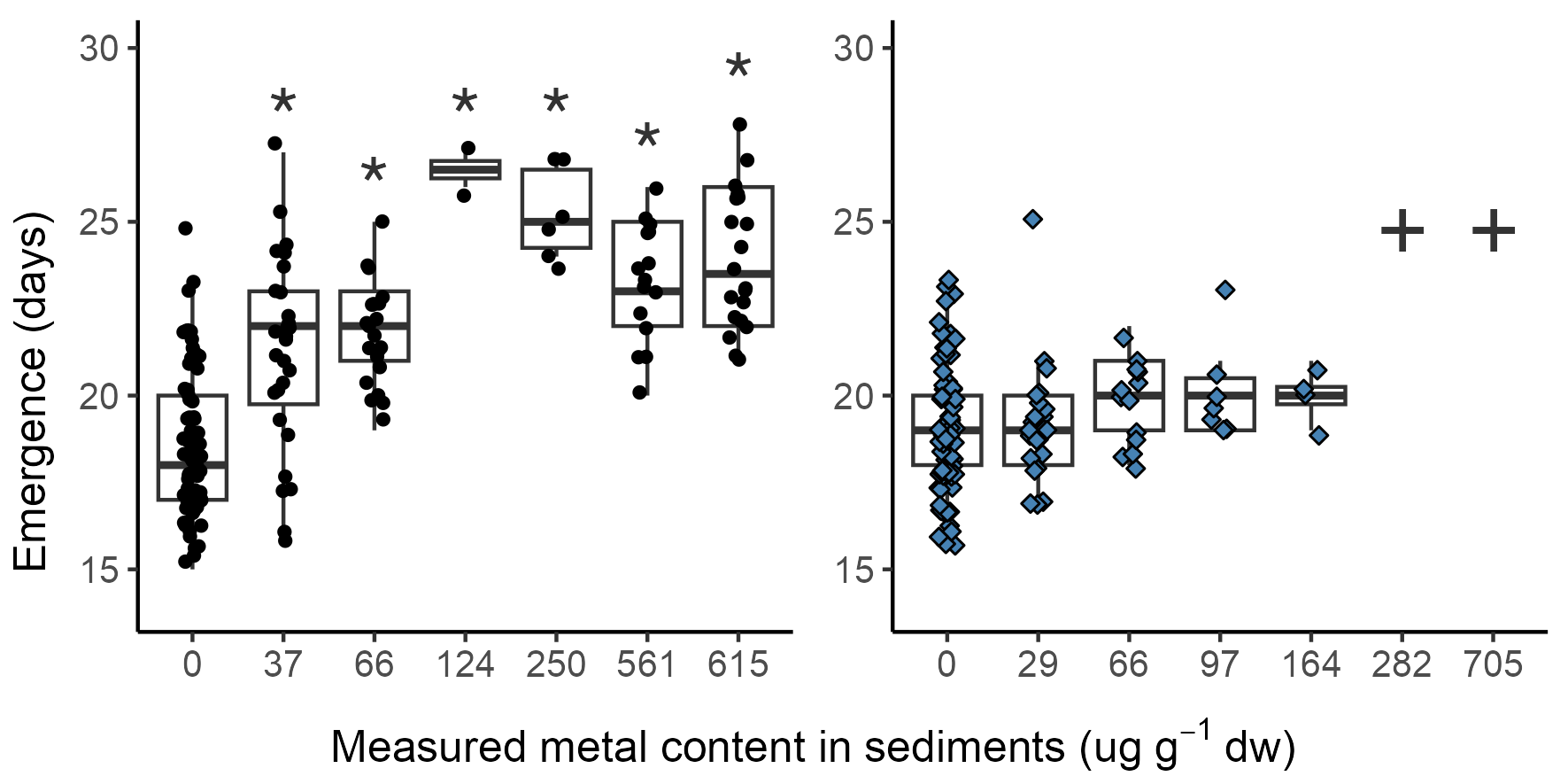


**Pd Pt**

| Parameter | Concentration (ug.g^-1^dw) | |
| --- | --- | --- |
|  | Pd | Pt |
| NOEC | < 37 | ND |
| LOEC | 37 | > 705 |

**Figure SI.9** Emergence (days) as a function of the measured content (μg_metal_.g_sed_-^1^ dw) of the emerged *C. riparius* for Pd (black points) and Pt (blue rhombuses). Toxicological parameters (NOEC; LOEC) are shown in the table below. The cross symbol (✚) denotes the absence of data at this content due to complete mortality of the exposed organisms. Asterisks (*) denote significant differences compared to control condition (p ≤ 0.05, n=4). ND: not determined.

**
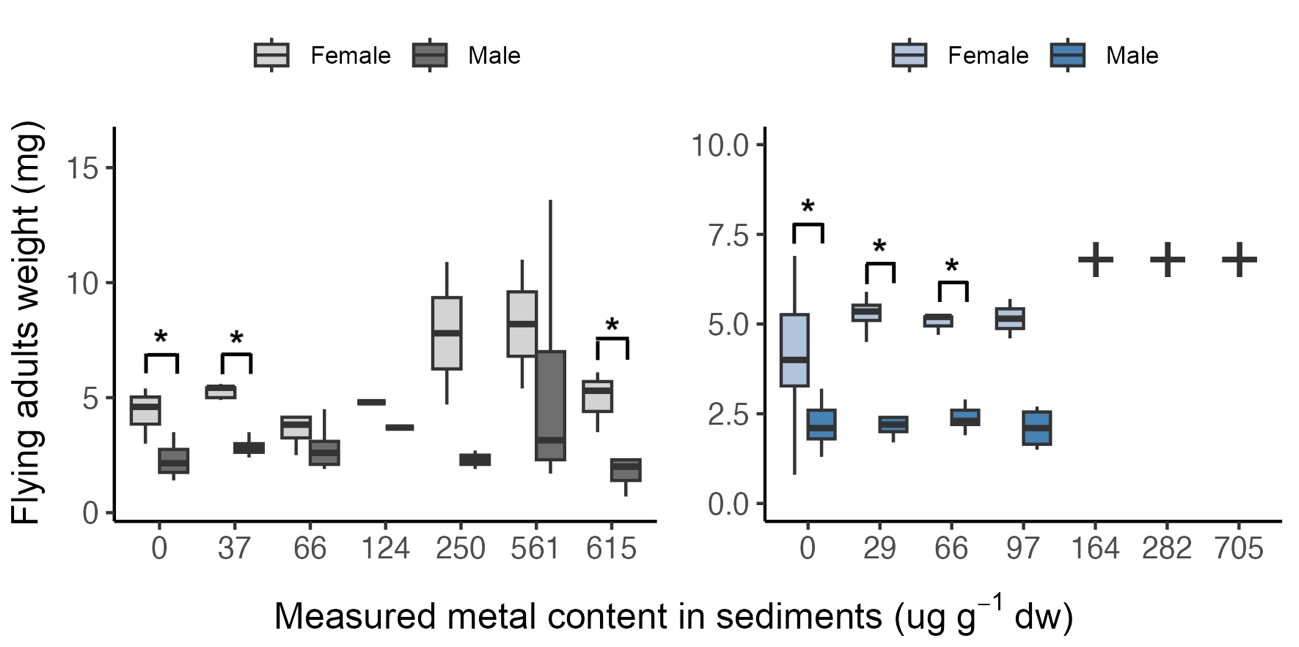
**

**Pd Pt**

**Figure SI.10** Flying adult weight (mg) as a function of the measured concentration (μg_metal_.g_sed_-^1^) of the emerged *C. riparius* for Pd (black points) and Pt (blue rhombuses). The cross symbol (✚) denotes the absence of data at this content due to complete mortality of the exposed organisms. A Kruskall-Wallis test denoted no significative impact of metal content on adult weight. A Wilcoxon–Mann–Whitney test showed significative differences between female and male weight for conditions shown by an asterisk (*).

**Table SI.1** Characterization of trace element composition and various parameters in tested sediments (50% of natural sediment from the Chaudière River (Quebec, Canada); 50% Carib Sea Super Natural Moonlight Sand artificial sediment).

| Parameter | | Unit | Value |
| --- | --- | --- | --- |
| Granulometry-Sand | | % | 66.3 |
| Granulometry-Lime | |  | 32.5 |
| Granulometry-Clay | |  | 6.6 |
| Total Organic Carbon | |  | 0.5 |
| Weight loss at 105 °C | |  | 24.8 |
| Sulfur | | µg.g^-1^ | NF |
| Petroleum hydrocarbons (C10-C50) | |  | 2.5 |
| Sum of PAHs and alkylated PAHs | |  | 2.0 |
| Organophosphate pesticides and other chemical groups | |  | <LDM |
| Glyphosate | |  | <LDM |
| Aminomethilphosphonic acid (AMPA) | |  | <LDM |
|  | |  |  |
| Trace element | Full Name | Detection Limit | Value |
|  |  | µg.g^-1^ dw | |
| Li | Lithium | 0.408 | 3.05 |
| Be | Beryllium | 0.241 | 0.07 |
| Na | Sodium | 10.42 | 33 |
| Mg | Magnesium | 10.79 | 855 |
| Al | Aluminum | 1.00 | 2690 |
| S | Sulfur | 10.98 | 16.3 |
| K | Potassium | 10.35 | 432 |
| Ca | Calcium | 10.14 | 581 |
| Cr | Chromium | 0.010 | 11.4 |
| Mn | Manganese | 0.008 | 68.6 |
| Co | Cobalt | 0.005 | 1.35 |
| Ni | Nickel | 0.011 | 4.48 |
| Sc | Scandium | 0.009 | 0.81 |
| Cu | Copper | 0.011 | 1.17 |
| Ti | Titanium | 0.100 | 217 |
| Zn | Zinc | 0.570 | 6.74 |
| V | Vanadium | 0.005 | 7.36 |
| Fe | Iron | 0.039 | 4452 |
| Ga | Gallium | 0.0021 | 1.01 |
| Rb | Rubidium | 0.0066 | 2.33 |
| Sr | Strontium | 0.0081 | 7.62 |
| As | Arsenic | 0.0048 | 0.96 |
| Se | Selenium | 0.0092 | 0.05 |
| Mo | Molybdenum | 0.0075 | 0.12 |
| Y | Yttrium | 0.0008 | 5.24 |
| Ru | Ruthenium | 0.0042 | <0.00 |
| Rh | Rhodium | 0.0013 | <0.00 |
| **Pd** | **Palladium** | **0.0012** | **0.00006** |
| Ag | Silver | 0.0050 | 0.01 |
| Cd | Cadmium | 0.0078 | 0.02 |
| Sb | Antimony | 0.0083 | 0.02 |
| Cs | Cesium | 0.0014 | 0.13 |
| Ba | Barium | 0.0017 | 14.59 |
| La | Lanthanum | 0.0005 | 22.56 |
| Ce | Cerium | 0.0003 | 48.14 |
| Pr | Praseodymium | 0.0004 | 5.65 |
| Nd | Neodymium | 0.0007 | 21.31 |
| Sm | Samarium | 0.0015 | 4.08 |
| Eu | Europium | 0.0009 | 0.35 |
| Gd | Gadolinium | 0.0008 | 3.04 |
| Yb | Ytterbium | 0.0011 | 0.30 |
| Tb | Terbium | 0.0004 | 0.34 |
| Dy | Dysprosium | 0.0008 | 1.49 |
| Ho | Holmium | 0.0006 | 0.22 |
| Er | Erbium | 0.0007 | 0.46 |
| Tm | Thulium | 0.0004 | 0.05 |
| Lu | Lutetium | 0.0004 | 0.04 |
| **Pt** | **Platinum** | **0.0055** | **0.0002** |
| Hg | Mercury | 0.0089 | 0.002 |
| Tl | Thallium | 0.0074 | 0.02 |
| Pb | Lead | 0.0081 | 3.03 |
| Th | Thorium | 0.0621 | 9.25 |
| U | Uranium | 0.0065 | 1.32 |

**Table SI.2** Values (mean ± SD; n=28 for the controls and n=81 for the conditions) of the physico-chemical parameters (pH, temperature, dissolved oxygen, conductivity, nitrites, nitrates and ammonium levels) in the water during the beginning, middle and end of acute and chronic tests.

|  | **pH** | **Temperature** | **O2** | **Conductivity** | **Ammonium** | **Nitrites** | **Nitrates** |  |
| --- | --- | --- | --- | --- | --- | --- | --- | --- |
|  |  | **°C** | **mg.L^-1^** | **µS.cm-^1^** | **mg.L^-1^** | | |  |
| **Acute *H. azteca* Pt** | | | | | | | | |
| Controls | 7.71 ± 0.14 | 23.24 ± 0.68 | 8.60 ± 0.10 | 433 ± 180 | 0.26 ± 0.39 | 0.44 ± 0.62 | 2.89 ± 4.18 |  |
| Conditions | 7.70 ± 0.10 | 23.55 ± 0.25 | 8.56 ± 0.08 | 522 ± 83 | 0.41 ± 0.48 | 0.30 ± 0.41 | 4.40 ± 6.58 |  |
| **Acute *H. azteca* Pd** | | | | | | | | |
| Controls | 7.63 ± 0.13 | 23.97 ± 0.20 | 8.64 ± 0.10 | 410 ± 34 | 0.13 ± 0.23 | 0.68 ± 0.90 | 2.00 ± 2.49 |  |
| Conditions | 7.41 ± 0.27 | 24.06 ± 0.22 | 8.63 ± 0.08 | 397 ± 19 | 0.86 ± 1.35 | 0.05 ± 0.21 | 0.89 ± 2.59 |  |
| **Acute *C. riparius* Pt** | | | | | | | | |
| Controls | 7.88 ± 0.08 | 20.35 ± 0.19 | 9.09 ± 0.07 | 306 ± 31 | 0.38 ± 0.45 | 0.13 ± 0.21 | 2.50 ± 5.37 |  |
| Conditions | 7.87 ± 0.06 | 20.51 ± 0.13 | 9.09 ± 0.11 | 293 ± 30 | 0.43 ± 0.41 | 0.19 ± 0.30 | 1.29 ± 2.12 |  |
| **Acute *C. riparius* Pd** | | | | | | | | |
| Controls | 7.79 ± 0.14 | 20.72 ± 0.14 | 9.03 ± 0.17 | 308 ± 28 | 0.41 ± 0.62 | 0.32 ± 0.60 | 1.01 ± 1.23 |  |
| Conditions | 7.68 ± 0.19 | 20.84 ± 0.18 | 9.03 ± 0.17 | 292 ± 21 | 0.10 ± 0.16 | 0.00 ± 0.03 | 0.11 ± 0.49 |  |
| **Chronic *C. riparius* Pt** | | | | | | | | |
| Controls | 8.03 ± 0.20 | 20.53 ± 0.14 | 9.14 ± 0.14 | 318 ± 34 | 0.12 ± 0.17 | 0.02 ± 0.05 | 0.57 ± 1.46 |  |
| Conditions | 7.99 ± 0.20 | 20.45 ± 0.23 | 9.17 ± 0.16 | 306 ± 43 | 0.10 ± 0.15 | 0.09 ± 0.28 | 0.82 ± 2.03 |  |
| **Chronic *C. riparius* Pd** | | | | | | | | |
| Controls | 7.92 ± 0.15 | 20.59 ± 0.16 | 9.12 ± 0.13 | 277 ± 25 | 0.07 ± 0.11 | 0.02 ± 0.07 | 1.21 ± 2.45 |  |
| Conditions | 7.95 ± 0.14 | 20.68 ± 0.19 | 9.09 ± 0.22 | 278 ± 25 | 0.11 ± 0.17 | 0.00 ± 0.00 | 0.04 ± 0.32 |  |

**Table SI.3** Recovery percentages (mean ± standard deviation) of platinum and palladium in the certified reference materials (CRMs), and the spiked materials after ICP-QQQ analysis (e.g. matrice or CRMs (+ spiked quantity, ng.g^-1^)).

|  | **Palladium** | | **Platinum** | | | |
| --- | --- | --- | --- | --- | --- | --- |
| **Organisms** |  |  |  |  |  |  |
| Reference material | IAEA-450 (+0.01)^a^ | IAEA-450 (+0.1)^a^ | IAEA-450 | | IAEA-450 (+0.01)^a^ | IAEA-450 (+ 0.1)^a^ |
| Mean ± standard deviation | 135* | 108 ± 13 | 100 ± 18 | | 134 ± 62 | 108 ± 9 |
| n | 8 | 8 | 20 | | 8 | 8 |
| **Sediments** |  |  |  | |  |  |
| Reference material | Control sediment (+40)^b^ | | Control sediment (+40)^b^ | | | |
| Mean ± standard deviation | 99 ± 9 | | 91 ± 10 | | | |
| n | 22 | | 22 | | | |

^*^Missing values due to unusable recoveries with high relative standard deviation values.

^a:^ For biological samples, accuracy and precision were assessed using the certified reference material IAEA-450 spiked with Pd and Pt standard solutions at 0.01 or 0.1 ng·g⁻¹.

^b:^ For sediment samples, measurement quality was verified using control sediments spiked with Pd and Pt standard solutions at 39,960 ng·g⁻¹.

**Table SI.4** Metal content (mean ± standard deviation, μg_metal_.g_sed_-^1^) in the metal-contaminated sediments at the beginning and the end of exposures for each test compared to the nominal content.

| Specie | Metal | Exposure duration | Nominal  [Metal] | Measured [Metal] in sediment | | | | Ratio [Measured] / [Nominal] |
| --- | --- | --- | --- | --- | --- | --- | --- | --- |
|  |  |  |  | Beginning of test | n | End of test | n |  |
|  |  |  |  | Mean ± standard deviation |  | Mean ± standard deviation |  | Beginning of test |
| *H. azteca* | Pd* | 14 | µg.g^-1^ dw | |  | µg.g^-1^ dw |  | % |
|  |  |  | 100 | 74 ± 3 | 2 | 67 ± 10 | 2 | 74 |
|  |  |  | 200 | 172 ± 13 | 2 | 130 ± 35 | 4 | 86 |
|  |  |  | 400 | 326 ± 80 | 2 | 223 ± 26 | 4 | 81 |
|  |  |  | 800 | 438 ± 21 | 2 | 365 ± 18 | 2 | 55 |
|  |  |  | 1600 | 804 ± 71 | 2 | 702 ± 62 | 2 | 50 |
|  |  |  | 2000 | 995 ± 59 | 2 | 902 ± 203 | 2 | 50 |
|  | Pt | 14 | 100 | 50 ± 11 | 2 | 20 ± 2 | 4 | 50 |
|  |  |  | 200 | 107 ± 12 | 2 | 81 ± 26 | 4 | 54 |
|  |  |  | 400 | 250 ± 101 | 2 | 169 ± 52 | 2 | 63 |
|  |  |  | 800 | 363 ± 50 | 2 | 465 ± 1 | 2 | 45 |
|  |  |  | 1600 | 1002 ± 108 | 2 | 614 ± - | 1 | 63 |
|  |  |  | 2000 | 1214 ± 147 | 2 | 1211 ± - | 1 | 61 |
| *C. riparius* | Pd* | 10 | 100 | 74 ± 3 | 2 | 67 ± 10 | 2 | 74 |
|  |  |  | 200 | 172 ± 13 | 2 | 130 ± 35 | 4 | 86 |
|  |  |  | 400 | 326 ± 80 | 2 | 223 ± 26 | 4 | 81 |
|  |  |  | 800 | 438 ± 21 | 2 | 365 ± 18 | 2 | 55 |
|  |  |  | 1600 | 804 ± 71 | 2 | 702 ± 62 | 2 | 50 |
|  |  |  | 2000 | 995 ± 59 | 2 | 902 ± 203 | 2 | 50 |
|  | Pt | 10 | 50 | 22 ± 5 | 2 | 20 ± 3 | 2 | 44 |
|  |  |  | 100 | 48 ± 4 | 2 | 19 ± 1 | 2 | 48 |
|  |  |  | 150 | 57 ± 13 | 2 | 67 ± 47 | 4 | 38 |
|  |  |  | 200 | 100 ± 47 | 2 | 63 ± 34 | 4 | 50 |
|  |  |  | 250 | 108 ± 84 | 2 | 76 ± 23 | 4 | 43 |
|  |  |  | 500 | 232 ± 4 | 2 | 183 ± 130 | 2 | 46 |
|  | Pd | 28 | 50 | 37 ± 5 | 2 | 40 ± 5 | 4 | 74 |
|  |  |  | 100 | 66 ± 7 | 2 | 74 ± 6 | 4 | 66 |
|  |  |  | 200 | 124 ± 21 | 2 | 146 ± 23 | 4 | 62 |
|  |  |  | 400 | 250 ± 14 | 2 | 293 ± 29 | 4 | 63 |
|  |  |  | 750 | 561 ± 31 | 2 | 485 ± 40 | 4 | 75 |
|  |  |  | 1000 | 615 ± 76 | 2 | 465 ± 61 | 4 | 61 |
|  | Pt | 28 | 50 | 29 ± 0 | 2 | 25 ± 8 | 2 | 58 |
|  |  |  | 100 | 66 ± 7 | 2 | 39 ± 12 | 4 | 66 |
|  |  |  | 150 | 97 ± - | 1 | 116 ± 99 | 4 | 65 |
|  |  |  | 200 | 164 ± 31 | 2 | 186 ± 1 | 2 | 82 |
|  |  |  | 400 | 282 ± - | 1 | 279 ± 248 | 2 | 70 |
|  |  |  | 1000 | 705 ± 39 | 2 | 550 ± - | 1 | 70 |

*Measured Pd concentrations during the 14-day exposure of *H. azteca* were assumed to be equivalent to those from the 10-day exposure of *C. riparius*, as both experiments involved identically prepared and handled sediments. Due to high variability in the measured values from the *H. azteca* test, likely stemming from inconsistent sediment mixing and sampling procedures, the more stable concentrations from the *C. riparius* exposure were used for interpretations.

**Table SI.5** Toxicological parameters derived from concentration–response curves for *Hyalella azteca* and *Chironomus riparius* exposed to Pd- and Pt-contaminated sediments during 10- and 14-days, respectively.

| Metal | Parameter | Concentration ± standard error (ug.g^-1^dw) | |
| --- | --- | --- | --- |
|  |  | *Hylella azteca* | *Chironomus riparius* |
| Pd | LC_20_ | 433 ± 214 | 57 ± 23 |
|  | LC_50_ | 1192 ± 356 | 209 ± 44 |
| Pt | LC_20_ | 169 ± 44 | 52 ± 7 |
|  | LC_50_ | 289 ± 28 | 84 ± 7 |

**Table SI.6** Summary of toxicological parameters for relative growth rate (%) in *Hyalella azteca* and *Chironomus riparius* exposed to measured concentrations of Pd and Pt in sediments.

| Organism | Parameter | Concentration (ug.g^-1^dw) | |
| --- | --- | --- | --- |
|  |  | Pd | Pt |
| *Hylella azteca* | NOEC | 74 | 250 |
|  | LOEC | 172 | 363 |
| *Chironomus riparius* | NOEC | 438 | < 108 |
|  | LOEC | 804 | 232 |

**Table SI.7** Summary of toxicological parameters for bioaccumulation (μg_metal_.g_organism_-^1^ dw) in *Hyalella azteca* and *Chironomus riparius* exposed to measured concentrations of Pd and Pt in sediments.

| Organism | Parameter | Concentration (ug.g^-1^dw) | |
| --- | --- | --- | --- |
|  |  | Pd | Pt |
| *Hylella azteca* | NOEC | 74 | 107 |
|  | LOEC | 172 | 250 |
| *Chironomus riparius* | NOEC | 438 | 22 |
|  | LOEC | 804 | 48 |

**Table SI.8** Toxicological parameters derived from concentration–response curves for *Chironomus riparius* exposed to Pd- and Pt-contaminated sediments during 28-days.

|  | Parameter | Concentration ± standard error (ug.g^-1^dw) |
| --- | --- | --- |
| Pd | LC_20_ | 1 ± 4 |
|  | LC_50_ | 107 ± 110 |
| Pt | LC_20_ | 21 ± 4 |
|  | LC_50_ | 161 ± 28 |

**Table SI.9** Summary of Median Lethal Concentration (LC50) and Median Effective Concentration (EC50) values reported in the literature for toxicity of soil and water contaminated with palladium or platinum metals (Pd: palladium; Pt: platinum) on terrestrial and aquatic invertebrate species. Our values are reported at the end of the table.

| **Species** | **Endpoint** | **Biological**  **parameter** | **Pd** | **Pt** | **Unit** | **Contaminated medium** | **Exposure time** | **Reference** |
| --- | --- | --- | --- | --- | --- | --- | --- | --- |
| *Enchytraeus crypticus* | Reproduction | EC50 | 70 | 162 | mmol.L^-1^ | Soil | 28 days | (Havelkova et al., 2014) |
| *Caenorhabditis elegans* | Reproduction | EC50 | 10 - 100 | 497 | μg.L^-1^ | Culture water | 96 hours | (Schertzinger et al., 2017) |
|  | Growth | EC50 | Bell-shaped concentration response curves | 808 | μg.L^-1^ |  |  |  |
|  | Fertility | EC50 |  | 726 | μg.L^-1^ |  |  |  |
| *Hyalella azteca* | Mortalité | LC50 | > 1000 | 131 | μg.L^-1^ | Softwater | 7 days | (Borgmann et al., 2005) |
|  |  | LC50 | 570 | 221 | μg.L^-1^ | Hardwater | 7 days |  |
| *Chironomus riparius* | Mobility | EC50 | > 32 | - | mg.L^-1^ | Culture water | 96 hours | (Lüderwald et al., 2016) |
|  | Mobility | EC50 | > 32 | - | mg.L^-1^ |  | 6 days |  |
| *Daphnia magna* | Mobility | EC50 | > 32 | - | mg.L^-1^ |  | 96 hours |  |
|  |  | EC50 | 1,2 | - | mg.L^-1^ |  | 6 days |  |
|  | Mortality | LC50 | 14 | 157 | μg.L^-1^ |  | 48 hours | (Zimmermann et al., 2017) |
|  | Mobility | EC50 | 19 | 276 | μg.L^-1^ |  | 24 hours |  |
|  | Mobility | EC50 | 13 | 110 | μg.L^-1^ |  | 48 hours |  |
| *Asellus aquaticus* | Mortality rate | T | 47 | 34 | % |  | 24 hours | (Moldovan et al., 2001) |
|  | Bioaccumulation factor | Bf | 150 | 85 | - |  | 24 hours |  |
| *Hyalella azteca* | Mortalité | LC50 | 2626 | 520 | mg.kg^-1^ | Sediment | 14 days | Present study |
| *Chironomus riparius* | Mortalité | LC50 | 283 | 192 | mg.kg^-1^ |  | 10 days |  |
|  | Mortalité | LC50 | 164 | 75 | mg.kg^-1^ |  | 28 days |  |

**Table SI.10** Summary of palladium and platinum accumulation in several environmental compartments (sediment, soil, road dust, surface water) in environments located around operating mines (Table A) and along roads in several countries (Table B).

| **TABLE A** | | | |
| --- | --- | --- | --- |
| **Study place** | **Content (ng.g^-1^)** | | **Reference** |
|  | **Pd** | **Pt** |  |
| Earth's crust | 0.4 ^2^ | 1 à 5 ^1^ | ^1^(OMS-IPCS, 1991, 2002)  ^2^(Sassani & Shock, 1998) |
| **Sediment** | | | |
| Tantaré lake, Quebec | 2 | 2.9 | (Bérubé, 2005) |
| Vose lake, Quebec | < 0.3 | 2.7 | (Bérubé, 2005) |
| Lac des Îles, Ontario | 0.3 – 90.8 | 0.3 – 14.2 | (Dyer & Russell, 2002) |
| Tulameen, British Columbia | 2 – 48 | 8 – 91 | (S. J. Cook & Fletcher, 1993) |
| **Soil** | | | |
| Sheen lake, Quebec | < 0.5 – 29 | < 2 – 306 | (N. J. Cook et al., 1992) |
| Bushveld complex, South Africa | – | 698 ± 178 | (Rauch & Fatoki, 2013) |
| Stillwater complex, Montana | 120 – 150 | 120 – 150 | (Fuchs & Rose, 1974) |
|  | Max : 725 | Max : 1 010 | (Riese & Arp, 1986) |
| Tulameen, British Columbia | 2 – 48 | 4 – 85 | (S. J. Cook & Fletcher, 1993) |

| **TABLE B** | | | |
| --- | --- | --- | --- |
| **Study place** | **Content** | | **Reference** |
|  | **Pd** | **Pt** |  |
| **Sediment (ng.g^-1^)** | | | |
| Boston Harbor, United States | 2.4 – 6.1 | 3.1 – 4.4 | (Tuit et al., 2000) |
| Pra River, Ghana, West Africa | < 10 – 537 | < 10 – 171 | (Essumang, 2008) |
| Shire Brook, England | 4 – 57 | 3 – 64 | (Prichard et al., 2008) |
| Rivière Humber, England | 5 – 8 | 6 – 8 | (Prichard et al., 2008) |
| Rivières Trent et Don, England | 2 – 14 | 2 – 35 | (Prichard et al., 2008) |
| Rivière Stour, England | < 0.16 – 12.8 | < 0.29 – 34.2 | (De Vos et al., 2002) |
| Avondale Creek, Australia | 12 ± 4 | 12 ± 3 | (Pratt & Lottermoser, 2007) |
| Toulon Bay, France | – | 6 – 15 | (Abdou et al., 2019) |
| Sediment of St-Lawrence  River, Canada | < DL | < DL | (Bluteau et al., 2025) |
| Suspended sediments of  St-Lawrence River, Canada | 0 - 3.6 | 0 - 23 | (Bluteau et al., 2025) |
| Ponds of Montreal, Canada | <DL - 22 | <DL - 12 | (Bluteau et al., 2025) |
| **Soil (ng.g^-1^)** | | | |
| Toronto, Canada | n.d. – 664 | 0,7 – 170 | (Wiseman et al., 2016) |
| United States | 18 – 31 | 64 – 73 | (Ely et al., 2001) |
| Karlsruhe, Germany | – | 154.9 ± 9.5 | (Fliegel et al., 2004) |
|  | n.d. – 27.2 | 112 – 166 | (Schäfer et al., 1999) |
| **Road dust (ng.g^-1^)** | | | |
| Toronto, Canada | 89 | 99 | (Wiseman et al., 2018) |
| Tunnels in Houston, United States | 770 ± 208 | 529 ± 130 | (Spada et al., 2012) |
| Roads in Houston, United States | 10 – 88 | 35 – 131 | (Spada et al., 2012) |
| Göterborg, Sweden | 70.8 | 325.5 | (Gómez et al., 2002) |
| Montreal, Canada | 0 - 354 | 0 - 130 | (Bluteau et al., 2025) |
| **Surface water (ng.L^-1^)** | | | |
| Thohoyandou, South Africa | n.d. – 26 100 | n.d. – 80 400 | (Odiyo et al., 2005) |
| Lorette River, Canada | < 1.2 – 39 | 0,1 – 0,4 | (Roy, 2009) |
| Duberger River, Canada | < 1.2 – 74 | < 0.3 – 1.1 | (Roy, 2009) |
| Rainwater pipes, Canada | < 1.2 – 830,1 | < 0.3 – 8.8 | (Roy, 2009) |

DL = Detection Limit

**Table SI.11** Summary of Median Lethal Concentration (LC50) and Median Effective Concentration (EC50) values reported in the literature for toxicity of sediment contact test contaminated with class-B and borderline metals (Cu: copper; Ni: nickel; U: Uranium; Hg: mercury; Pb: lead; Zn: zinc; Cd: cadmium; As: arsenic; Ag: silver; Mo: Molybdène) on *Chironomus riparius* or *dilitus* and *Hyaella azteca* species.

| **Metal** | **Species** | **Endpoint** | **LC50/EC50** | **Value** | **Unit** | **Reference** | **Exposure time** |
| --- | --- | --- | --- | --- | --- | --- | --- |
| Cu | *C. riparius* | Mortality | LC50 | 0.033 | mmol/dm³ | (Bechard et al., 2008) | 24 hours |
|  |  |  | LC50 | 0.211 | mmol/dm³ | (Hooftman et al., 1989) | 24 hours |
|  |  |  | LC50 | 0.001 | mmol/dm³ | (Milani et al., 2003) | 96 hours |
|  |  |  | LC50 | 402 | mg.kg^-1^ | (Milani et al., 2003) | 10 days |
|  |  |  | LC50 | 320 | mg.kg^-1^ | (Roman et al., 2007) | 14 days |
|  |  | Growth | EC50 | 150 | mg.kg^-1^ | (Roman et al., 2007) | 28 days |
|  |  | Emergence | EC50 | 59,2 | mg.kg^-1^ | (Roman et al., 2007) | 28 days |
|  | *H. azteca* | Mortality | LC50 | 128 | mg.kg^-1^ | (Milani et al., 2003) | 28 days |
|  |  |  | LC50 | 316 | mg.kg^-1^ | (Roman et al., 2007) | 14 days |
|  |  | Growth | EC50 | 194 | mg.kg^-1^ | (Roman et al., 2007) | 28 days |
| Ni | *C. riparius* | Mortality | LC50 | 665 | mg.kg^-1^ | (Milani et al., 2003) | 10 days |
|  |  | Emergence | EC50 | 146 | mg.kg^-1^ | (Jesus et al., 2022) | 28 days |
|  | *H. azteca* | Mortality | LC50 | 67 | mg.kg^-1^ | (Milani et al., 2003) | 28 days |
|  |  |  | LC50 | 521 | mg.kg^-1^ | (Liber et al., 2011) | 10 days |
|  |  | Growth | EC50 | 312 | mg.kg^-1^ | (Liber et al., 2011) | 10 days |
|  | *C. dilitus* | Mortality | LC50 | > 3286 | mg.kg^-1^ | (Liber et al., 2011) | 10 days |
|  |  | Growth | EC50 | 1281 | mg.kg^-1^ | (Liber et al., 2011) | 10 days |
| U | *H. azteca* | Mortality | LC50 | 2442 | mg.kg^-1^ | (Liber et al., 2011) | 10 days |
|  |  | Growth | EC50 | 1918 | mg.kg^-1^ | (Liber et al., 2011) | 10 days |
|  | *C. riparius* | Mortality | LC50 | 5.3 | mg.kg^-1^ | (Dias et al., 2008) | 10 days |
| Hg | *C. riparius* | Mortality | LC50 | 0.016 | mmol.dm^-^³ | (Hooftman et al., 1989) | 24 hours |
|  |  |  | LC50 | 0.005 | mmol. dm^-^³ | (Rossaro et al., 1986) | 24 hours |
| Pb | *C. riparius* | Mortality | LC50 | 0.0029 | mmol. dm^-^³ | (Bechard et al., 2008) | 24 hours |
|  |  |  | LC50 | 0.0035 | mmol. dm^-^³ | (Timmermans et al., 1992) | 32 hours |
|  | *H. azteca* | Mortality | LC50 | 35 | µmol.g^-1^ | (Borgmann & Norwood, 1999) | 4 weeks |
| Zn | *C. riparius* | Mortality | LC50 | >0.382 | mmol. dm^-^³ | (Bechard et al., 2008) | 24 hours |
|  |  |  | LC50 | 1.442 | mmol. dm^-^³ | (Ibrahim et al., 1998) | 24 hours |
| Cd | *C. riparius* | Mortality | LC50 | 0.083 | mmol. dm^-^³ | (Bechard et al., 2008) | 24 hours |
|  |  |  | LC50 | 0.019 | mmol. dm^-^³ | (Williams et al., 1986) | 24 hours |
|  |  |  | LC50 | 39 | mg.kg^-1^ | (Milani et al., 2003) | 10 days |
|  |  |  | LC50 | 172,66 | mg.kg^-1^ | (Z. Liu et al., 2021) | 21 days |
|  |  | Growth | EC50 | 1,5 | mg.kg^-1^ | (Péry et al., 2008) | 10 days |
|  | *H. azteca* | Mortality | LC50 | 33 | mg.kg^-1^ | (Milani et al., 2003) | 28 days |
| As | *C. dilutus* | Mortality | LC50 | 642 | mg.kg^-1^ | (Liber et al., 2011) | 10 days |
|  | *H. azteca* | Mortality | LC50 | 532 | mg/kg | (Liber et al., 2011) | 10 days |
|  |  |  | LC50 | >462 | mg/kg | (Liber et al., 2011) | 10 days |
| Ag | *C. tentans* | Mortality | LC50 | 1.96 | mg.kg^-1^ | (Call et al., 1999) | 10 days |
|  | *H. azteca* | Mortality | LC50 | 0.084 | g.kg^-1^ | (Call et al., 2006) | 10 days |
| Mo | *H. azteca* | Mortality | LC50 | >3742 | mg.kg^-1^ | (Liber et al., 2011) | 10 days |
|  |  | Growth | EC50 | >3742 | mg.kg^-1^ | (Liber et al., 2011) | 10 days |
| Pd | *C. riparius* | Mortality | LC50 | 209 | mg.kg^-1^ | Present study | 10 days |
|  |  | Mortality | LC50 | 107 | mg.kg^-1^ | Present study | 28 days |
|  | *H. azteca* | Mortality | LC50 | 1192 | mg.kg^-1^ | Present study | 14 days |
| Pt | *C. riparius* | Mortality | LC50 | 84 | mg.kg^-1^ | Present study | 10 days |
|  |  | Mortality | LC50 | 161 | mg.kg^-1^ | Present study | 28 days |
|  | *H. azteca* | Mortality | LC50 | 289 | mg.kg^-1^ | Present study | 14 days |

**Supplemental references**

Abdou, M., Schäfer, J., Hu, R., Gil-Díaz, T., Garnier, C., Brach-Papa, C., Chiffoleau, J.-F., Charmasson, S., Giner, F., Dutruch, L., & Blanc, G. (2019). Platinum in sediments and mussels from the northwestern Mediterranean coast : Temporal and spatial aspects. *Chemosphere*, *215*, 783‑792. https://doi.org/10.1016/j.chemosphere.2018.10.011

Bechard, K. M., Gillis, P. L., & Wood, C. M. (2008). Acute Toxicity of Waterborne Cd, Cu, Pb, Ni, and Zn to First-Instar *Chironomus riparius* Larvae. *Archives of Environmental Contamination and Toxicology*, *54*(3), 454‑459. https://doi.org/10.1007/s00244-007-9048-7

Bérubé, L. (2005). *Mobilité et géochronologie du Pt et du Pd dans les sédiments de deux lacs du Québec* [Doctoral dissertation, Université du Québec, Institut national de la recherche scientifique]. https://espace.inrs.ca/id/eprint/395/

Bluteau, G., Ponton, D. E., Rosabal, M., & Amyot, M. (2025). Biodynamics and Environmental Concentrations of the Platinum Group Elements in Freshwater Systems. *Environmental Science & Technology*, acs.est.4c08750. https://doi.org/10.1021/acs.est.4c08750

Borgmann, U., Couillard, Y., Doyle, P., & Dixon, D. G. (2005). Toxicity of sixty-three metals and metalloids to *Hyalella azteca* at two levels of water hardness. *Environmental Toxicology and Chemistry*, *24*(3), 641‑652. https://doi.org/10.1897/04-177R.1

Borgmann, U., & Norwood, W. P. (1999). Assessing the toxicity of lead in sediments to *Hyalella azteca* : The significance of bioaccumulation and dissolved metal. *Canadian Journal of Fisheries and Aquatic Sciences*, *56*(8), 1494‑1503. https://doi.org/10.1139/f99-073

Cairns, M. A., Nebeker, A. V., Gakstatter, J. H., & Griffis, W. L. (1984). Toxicity of copper-spiked sediments to freshwater invertebrates. *Environmental Toxicology and Chemistry*, *3*(3), 435‑445. https://doi.org/10.1002/etc.5620030308

Call, D. J., Polkinghorne, C. N., Markee, T. P., Brooke, L. T., Geiger, D. L., Gorsuch, J. W., & Robillard, K. A. (1999). Silver toxicity to *Chironomus tentans* in two freshwater sediments. *Environmental Toxicology and Chemistry*, *18*(1), 30‑39. https://doi.org/10.1002/etc.5620180105

Call, D. J., Polkinghorne, C. N., Markee, T. P., Brooke, L. T., Geiger, D. L., Gorsuch, J. W., & Robillard, K. A. (2006). Toxicity of silver in water and sediment to the freshwater amphipod *Hyalella azteca*. *Environmental Toxicology and Chemistry*, *25*(7), 1802‑1808. https://doi.org/10.1897/05-319R.1

Carney Almroth, B., Carle, A., Blanchard, M., Molinari, F., & Bour, A. (2023). Single-use take-away cups of paper are as toxic to aquatic midge larvae as plastic cups. *Environmental Pollution*, *330*, 121836. <https://doi.org/10.1016/j.envpol.2023.121836>

CEAEQ. *Entretien de l’élevage de* Chironomus riparius (DR-09-BMS-027). Centre d’expertise en analyse environnementale du Québec.

Cook, N. J., Wood, S. A., & Zhang, Y. (1992). Transport and fixation of Au, Pt and Pd around the Lac Sheen Cu-Ni-PGE occurrence in Quebec, Canada. *Journal of Geochemical Exploration*, *46*(2), 187‑228. https://doi.org/10.1016/0375-6742(92)90079-N

Cook, S. J., & Fletcher, W. K. (1993). Distribution and behaviour of platinum in soils, sediments and waters of the Tulameen ultramafic complex, southern British Columbia, Canada. *Journal of Geochemical Exploration*, *46*(3), 279‑308. https://doi.org/10.1016/0375-6742(93)90026-I

De Vos, E., Edwards, S. J., McDonald, I., Wray, D. S., & Carey, P. J. (2002). A baseline survey of the distribution and origin of platinum group elements in contemporary fluvial sediments of the Kentish Stour, England. *Applied Geochemistry*, *17*(8), 1115‑1121. https://doi.org/10.1016/S0883-2927(02)00010-0

Dias, V., Vasseur, C., & Bonzom, J.-M. (2008). Exposure of Chironomus riparius larvae to uranium : Effects on survival, development time, growth, and mouthpart deformities. *Chemosphere*, *71*(3), 574‑581. <https://doi.org/10.1016/j.chemosphere.2007.09.029>

Di Toro, D. M., Mahony, J. D., Hansen, D. J., Scott, K. J., Hicks, M. B., Mayr, S. M., & Redmond, M. S. (1990). Toxicity of cadmium in sediments : The role of acid volatile sulfide. *Environmental Toxicology and Chemistry*, *9*(12), 1487‑1502. <https://doi.org/10.1002/etc.5620091208>

Dyer, R. D., & Russell, D. F. (2002). Lac des Iles–Black Sturgeon River area lake sediment survey : Operation Treasure Hunt (Ontario Geological Survey, Open File Report 6096, p. 134).

Ely, J. C., Neal, C. R., Kulpa, C. F., Schneegurt, M. A., Seidler, J. A., & Jain, J. C. (2001). Implications of Platinum-Group Element Accumulation along U.S. Roads from Catalytic-Converter Attrition. *Environmental Science & Technology*, *35*(19), 3816‑3822. https://doi.org/10.1021/es001989s

Environnement Canada. (1995). *Document d’orientation sur la mesure de la précision des essais de toxicité sur sédiment de contrôle dopé avec un produit toxique de référence* (0-660-95241‑6; p. 54). Ministre des Travaux publics et Services gouvernementaux Canada.

Environnement Canada. (1997). *Méthode d’essai biologique : Essai de survie et de croissance des larves dulcicoles de chironomes (*Chironomus tentans *ou* Chironomus riparius*) dans les sédiments* (SPE 1/RM/32; p. 156). Centre des sciences et technologies environnementales. <https://publications.gc.ca/collections/collection_2014/ec/En49-24-1-32-fra.pdf>

Environnement Canada. (2017). *Méthode d’essai biologique : Essai de survie, de croissance et de reproduction de l’amphipode dulcicole Hyalella azteca dans les sédiments et l’eau* (RM/33RM/33; p. 207). Centre des sciences et technologies environnementales. <https://publications.gc.ca/collections/collection_2018/eccc/En49-7-1-33-2017-fra.pdf>

Essumang, D. (2008). Bioaccumulation of platinum group metals in dolphins, *Stenella sp.*, caught off Ghana. *African Journal of Aquatic Science*, *33*(3), 255‑259. https://doi.org/10.2989/AJAS.2008.33.3.8.620

Fagin, D. (2012). Toxicology : The learning curve. *Nature*, *490*(7421), Article 7421. https://doi.org/10.1038/490462a

Fliegel, D., Berner, Z., Eckhardt, D., & Steben, D. (2004). New data on the mobility of Pt emitted from catalytic converters. *Analytical and Bioanalytical Chemistry*, *379*(1), 131‑136. https://doi.org/10.1007/s00216-004-2556-7

Fuchs, W. A., & Rose, A. W. (1974). The Geochemical Behavior of Platinum and Palladium in the Weathering Cycle in the Stillwater Complex, Montana. *Economic Geology*, *69*(3), 332‑346. https://doi.org/10.2113/gsecongeo.69.3.332

Georgescu, B., Georgescu, C., Dărăban, S., Bouaru, A., & Pașcalău, S. (2011). Heavy Metals Acting as Endocrine Disrupters. *SCIENTIFIC PAPERS ANIMAL SCIENCE AND BIOTECHNOLOGIES*, *44*(2), Article 2.

Gerhardt, A. (1993). Review of impact of heavy metals on stream invertebrates with special emphasis on acid conditions. *Water, Air, and Soil Pollution*, *66*(3), 289‑314. https://doi.org/10.1007/BF00479852

Gómez, B., Palacios, M. A., Gómez, M., Sanchez, J. L., Morrison, G., Rauch, S., McLeod, C., Ma, R., Caroli, S., Alimonti, A., Petrucci, F., Bocca, B., Schramel, P., Zischka, M., Petterson, C., & Wass, U. (2002). Levels and risk assessment for humans and ecosystems of platinum-group elements in the airborne particles and road dust of some European cities. *Science of The Total Environment*, *299*(1‑3), 1‑19. https://doi.org/10.1016/S0048-9697(02)00038-4

Havelkova, B., Kovacova, V., Bednarova, I., Pikula, J., & Beklova, M. (2014). Impact of platinum group elements on the soil invertebrate *Enchytraeus crypticus*. *Neuro Endocrinology Letters*, *35 Suppl 2*, 43‑50.

Hooftman, R. N., Adema, D. M. M., & Kauffman-Van Bommel, J. (1989). Developing a set of test methods for the toxicological analysis of the pollution degree of waterbottoms. *Netherlands Organization for Applied Scientific Research. Report*, *16105*.

Hourtané, O., Rioux, G., Campbell, P. G. C., & Fortin, C. (2022). Algal bioaccumulation and toxicity of platinum are increased in the presence of humic acids. *Environmental Chemistry*, *19*(4), 144‑155. https://doi.org/10.1071/EN22037

Hourtané, O., Smith, D. S., & Fortin, C. (2024). Natural organic matter (NOM) can increase the uptake fluxes of three critical metals (Ga, La, Pt) in a unicellular green alga. *Chemosphere*, *365*, 143311. https://doi.org/10.1016/j.chemosphere.2024.143311

Ibrahim, H., Kheir, R., Helmi, S., Lewis, J., & Crane, M. (1998). Effects of organophosphorus, carbamate, pyrethroid and organochlorine pesticides, and a heavy metal on survival and cholinesterase activity of *Chironomus riparius* Meigen. *Bulletin of Environmental Contamination and Toxicology*, *60*, 448‑455.

Jesus, F., Patrício Silva, A. L., Pereira, J. L., Ré, A., Campos, I., Gonçalves, F. J. M., Nogueira, A. J. A., Abrantes, N., & Serpa, D. (2022). Do sediment-bound nickel and lead affect chironomids life-history? Toxicity assessment under environmentally relevant conditions. *Aquatic Toxicology*, *253*, 106347. https://doi.org/10.1016/j.aquatox.2022.106347

Lacey, R., Watzin, M. C., & McIntosh, A. W. (1999). Sediment organic matter content as a confounding factor in toxicity tests with *Chironomus tentans*. *Environmental Toxicology and Chemistry*, *18*(2), 231‑236. https://doi.org/10.1002/etc.5620180219

Leopold, K., Wörle, K., Schindl, R., Huber, L., Maier, M., & Schuster, M. (2017). Determination of traffic-related palladium in tunnel dust and roadside soil. *Science of The Total Environment*, *583*, 169‑175. https://doi.org/10.1016/j.scitotenv.2017.01.048

Liber, K., Doig, L. E., & White-Sobey, S. L. (2011). Toxicity of uranium, molybdenum, nickel, and arsenic to *Hyalella azteca* and *Chironomus dilutus* in water-only and spiked-sediment toxicity tests. *Ecotoxicology and Environmental Safety*, *74*(5), 1171‑1179. https://doi.org/10.1016/j.ecoenv.2011.02.014

Liu, D., Shi, Q., Liu, C., Sun, Q., & Zeng, X. (2023). Effects of Endocrine-Disrupting Heavy Metals on Human Health. *Toxics*, *11*(4), Article 4. https://doi.org/10.3390/toxics11040322

Liu, Z., Zhang, C., Xin, Z., Tai, P., Song, C., & Deng, X. (2021). Comparing the Impacts of Sediment-Spiked Cadmium on Chironomidae Larvae in Laboratory Bioassays and Field Microcosms and the Implications for Field Validation of Site-Specific Threshold Concentrations. *Environmental Toxicology and Chemistry*, *40*(9), 2450‑2462. https://doi.org/10.1002/etc.5073

Lüderwald, S., Seitz, F., Seisenbaeva, G. A., Kessler, V. G., Schulz, R., & Bundschuh, M. (2016). Palladium Nanoparticles : Is There a Risk for Aquatic Ecosystems? *Bulletin of Environmental Contamination and Toxicology*, *97*(2), 153‑158. https://doi.org/10.1007/s00128-016-1803-x

Lustig, S., Zang, S., Beck, W., & Schramel, P. (1998). Dissolution of metallic platinum as water soluble species by naturally occurring complexing agents. *Microchimica Acta*, *129*(3), 189‑194. https://doi.org/10.1007/BF01244740

Mays, J. W. (2009). *Bioaccumulation of Platinum Group Metals in the Freshwater Mussel* Elliptio Complanata. http://www.lib.ncsu.edu/resolver/1840.16/2528

Milani, D., Reynoldson, T. B., Borgmann, U., & Kolasa, J. (2003). The relative sensitivity of four benthic invertebrates to metals in spiked-sediment exposures and application to contaminated field sediment. *Environmental Toxicology and Chemistry*, *22*(4), 845‑854. https://doi.org/10.1002/etc.5620220424

Moldovan, M., Rauch, S., Gómez, M., Antonia Palacios, M., & Morrison, G. M. (2001). Bioaccumulation of palladium, platinum and rhodium from urban particulates and sediments by the freshwater isopod *Asellus aquaticus*. *Water Research*, *35*(17), 4175‑4183. https://doi.org/10.1016/S0043-1354(01)00136-1

Nieboer, E., & Richardson, D. H. S. (1980). The replacement of the nondescript term ‘heavy metals’ by a biologically and chemically significant classification of metal ions. *Environmental Pollution Series B, Chemical and Physical*, *1*(1), 3‑26. https://doi.org/10.1016/0143-148X(80)90017-8

Nieto, E., Corada-Fernández, C., Hampel, M., Lara-Martín, P. A., Sánchez-Argüello, P., & Blasco, J. (2017). Effects of exposure to pharmaceuticals (diclofenac and carbamazepine) spiked sediments in the midge, *Chironomus riparius* (Diptera, Chironomidae). *Science of The Total Environment*, *609*, 715‑723. https://doi.org/10.1016/j.scitotenv.2017.07.171

Obata, H., Yoshida, T., & Ogawa, H. (2006). Determination of picomolar levels of platinum in estuarine waters : A comparison of cathodic stripping voltammetry and isotope dilution-inductively coupled plasma mass spectrometry. *Analytica Chimica Acta*, *580*(1), 32‑38. https://doi.org/10.1016/j.aca.2006.07.044

Odiyo, J. O., Bapela, H. M., Mugwedi, R., & Chimuka, L. (2005). Metals in environmental media : A study of trace and platinum group metals in Thohoyandou, South Africa. *Water SA*, *31*(4), Article 4. https://doi.org/10.4314/wsa.v31i4.5148

OMS-IPCS. (1991). *Environmental Health Criteria no125 : Platinum*. Genève, Organisation mondiale de la Santé, International Programme on Chemical Safety. [http://www.inchem.org/documents/ehc/ehc/ehc125.htm

OMS-IPCS. (2002). *Environmental Health Criteria 226 : Palladium*. Organisation mondiale de la Santé, International Program of Chemical Safety. https://www.inchem.org/documents/ehc/ehc/ehc226.htm

Paquin, P. R., Gorsuch, J. W., Apte, S., Batley, G. E., Bowles, K. C., Campbell, P. G. C., Delos, C. G., Di Toro, D. M., Dwyer, R. L., Galvez, F., Gensemer, R. W., Goss, G. G., Hogstrand, C., Janssen, C. R., McGeer, J. C., Naddy, R. B., Playle, R. C., Santore, R. C., Schneider, U., … Wu, K. B. (2002). The biotic ligand model : A historical overview. *Comparative Biochemistry and Physiology Part C: Toxicology & Pharmacology*, *133*(1), 3‑35. https://doi.org/10.1016/S1532-0456(02)00112-6

Péry, A. R. R., Geffard, A., Conrad, A., Mons, R., & Garric, J. (2008). Assessing the risk of metal mixtures in contaminated sediments on *Chironomus riparius* based on cytosolic accumulation. *Ecotoxicology and Environmental Safety*, *71*(3), 869‑873. https://doi.org/10.1016/j.ecoenv.2008.04.009

Pratt, C., & Lottermoser, B. G. (2007). Mobilisation of traffic-derived trace metals from road corridors into coastal stream and estuarine sediments, Cairns, northern Australia. *Environmental Geology*, *52*(3), 437‑448. https://doi.org/10.1007/s00254-006-0471-2

Prichard, H. M., Jackson, M. T., & Sampson, J. (2008). Dispersal and accumulation of Pt, Pd and Rh derived from a roundabout in Sheffield (UK) : From stream to tidal estuary. *Science of The Total Environment*, *401*(1‑3), 90‑99. https://doi.org/10.1016/j.scitotenv.2008.03.037

Rauch, S., & Fatoki, O. S. (2013). Anthropogenic Platinum Enrichment in the Vicinity of Mines in the Bushveld Igneous Complex, South Africa. *Water, Air, & Soil Pollution*, *224*(1), 1395. https://doi.org/10.1007/s11270-012-1395-y

Rauch, S., & Morrison, G. M. (1999). Platinum uptake by the freshwater isopod *Asellus Aquaticus* in urban rivers. *Science of The Total Environment*, *235*(1), 261‑268. https://doi.org/10.1016/S0048-9697(99)00203-X

Riese, W. C., & Arp, G. K. (1986). *Biogeochemical exploration for platinum deposits in the Stillwater Complex, Montana. In Mineral Exploration : Biological Systems and Organic Matter* (p. 170‑182).

Rioux, G. (2018). *Spéciation du platine en présence de matière organique naturelle et biodisponibilité pour l’algue verte Chlamydomonas reinhardtii.* [Masters, Université du Québec, Institut national de la recherche scientifique]. https://espace.inrs.ca/id/eprint/7624/

Roman, Y. E., De Schamphelaere, K. A. C., Nguyen, L. T. H., & Janssen, C. R. (2007). Chronic toxicity of copper to five benthic invertebrates in laboratory-formulated sediment : Sensitivity comparison and preliminary risk assessment. *Science of The Total Environment*, *387*(1), 128‑140. https://doi.org/10.1016/j.scitotenv.2007.06.023

Rossaro, B., Gaggino, G. F., & Marchetti, R. (1986). Accumulation of mercury in larvae and adults, *Chironomus riparis* (Meigen). *Bulletin of environmental contamination and toxicology*, *37*(3), 402‑406.

Roy, G. (2009). *Les éléments du groupe platine (Pd, Pt et Rh) dans les eaux de surface et leur toxicité chez l’algue verte Chlamydonomas reinhardtii* [Masters, Université du Québec, Institut national de la recherche scientifique]. https://espace.inrs.ca/id/eprint/482/

Saraiva, A. S., Sarmento, R. A., Gravato, C., Rodrigues, A. C. M., Campos, D., Simão, F. C. P., & Soares, A. M. V. M. (2020). Strategies of cellular energy allocation to cope with paraquat-induced oxidative stress : Chironomids vs Planarians and the importance of using different species. *Science of The Total Environment*, *741*, 140443. https://doi.org/10.1016/j.scitotenv.2020.140443

Sassani, D. C., & Shock, E. L. (1998). Solubility and transport of platinum-group elements in supercritical fluids : Summary and estimates of thermodynamic properties for ruthenium, rhodium, palladium, and platinum solids, aqueous ions, and complexes to 1000°C and 5 kbar. *Geochimica et Cosmochimica Acta*, *62*(15), 2643‑2671. https://doi.org/10.1016/S0016-7037(98)00049-0

Schäfer, J., Eckhardt, J.-D., Berner, Z. A., & Stüben, D. (1999). Time-Dependent Increase of Traffic-Emitted Platinum-Group Elements (PGE) in Different Environmental Compartments. *Environmental Science & Technology*, *33*(18), 3166‑3170. https://doi.org/10.1021/es990033i

Schertzinger, G., Zimmermann, S., Grabner, D., & Sures, B. (2017). Assessment of sublethal endpoints after chronic exposure of the nematode *Caenorhabditis elegans* to palladium, platinum and rhodium. *Environmental Pollution*, *230*, 31‑39. https://doi.org/10.1016/j.envpol.2017.06.040

Schindl, R., & Leopold, K. (2015). Analysis of Platinum Group Elements in Environmental Samples : A Review. In F. Zereini & C. L. S. Wiseman (Éds.), *Platinum Metals in the Environment* (p. 109‑128). Springer. https://doi.org/10.1007/978-3-662-44559-4_8

Singer, C., Zimmermann, S., & Sures, B. (2005). Induction of heat shock proteins (hsp70) in the zebra mussel (*Dreissena polymorpha*) following exposure to platinum group metals (platinum, palladium and rhodium) : Comparison with lead and cadmium exposures. *Aquatic Toxicology*, *75*(1), 65‑75. https://doi.org/10.1016/j.aquatox.2005.07.004

Spada, N., Bozlaker, A., & Chellam, S. (2012). Multi-elemental characterization of tunnel and road dusts in Houston, Texas using dynamic reaction cell-quadrupole-inductively coupled plasma–mass spectrometry : Evidence for the release of platinum group and anthropogenic metals from motor vehicles. *Analytica Chimica Acta*, *735*, 1‑8. https://doi.org/10.1016/j.aca.2012.05.026

Sures, B., & Zimmermann, S. (2007). Impact of humic substances on the aqueous solubility, uptake and bioaccumulation of platinum, palladium and rhodium in exposure studies with *Dreissena polymorpha*. *Environmental Pollution*, *146*(2), 444‑451. https://doi.org/10.1016/j.envpol.2006.07.004

Timmermans, K. R., Peeters, W., & Tonkes, M. (1992). Cadmium, zinc, lead and copper in *Chironomus riparis* (Meigen) larvae (Diptera, Chironomidae) : Uptake and effects. *Hydrobiologia*, *241*(2), 119‑134. https://doi.org/10.1007/BF00008264

Tuit, C. B., Ravizza, G. E., & Bothner, M. H. (2000). Anthropogenic Platinum and Palladium in the Sediments of Boston Harbor. *Environmental Science & Technology*, *34*(6), 927‑932. https://doi.org/10.1021/es990666x

Williams, K. A., Green, D. W. J., Pascoe, D., & Gower, D. E. (1986). The acute toxicity of cadmium to different larval stages of *Chironomus riparius* (Diptera : Chironomidae) and its ecological significance for pollution regulation. *Oecologia*, *70*(3), 362‑366. https://doi.org/10.1007/BF00379498

Wiseman, C. L. S., Hassan Pour, Z., & Zereini, F. (2016). Platinum group element and cerium concentrations in roadside environments in Toronto, Canada. *Chemosphere*, *145*, 61‑67. https://doi.org/10.1016/j.chemosphere.2015.11.056

Wiseman, C. L. S., Niu, J., Levesque, C., Chénier, M., & Rasmussen, P. E. (2018). An assessment of the inhalation bioaccessibility of platinum group elements in road dust using a simulated lung fluid. *Environmental Pollution*, *241*, 1009‑1017. https://doi.org/10.1016/j.envpol.2018.06.043

Zereini, F., Skerstupp, B., Alt, F., Helmers, E., & Urban, H. (1997). Geochemical behaviour of platinum-group elements (PGE) in particulate emissions by automobile exhaust catalysts : Experimental results and environmental investigations. *Science of The Total Environment*, *206*(2‑3), 137‑146. https://doi.org/10.1016/S0048-9697(97)80005-8

Zimmermann, S., Messerschmidt, J., von Bohlen, A., & Sures, B. (2005). Uptake and bioaccumulation of platinum group metals (Pd, Pt, Rh) from automobile catalytic converter materials by the zebra mussel (*Dreissena polymorpha*). *Environmental Research*, *98*(2), 203‑209. https://doi.org/10.1016/j.envres.2004.08.005

Zimmermann, S., Wolff, C., & Sures, B. (2017). Toxicity of platinum, palladium and rhodium to *Daphnia magna* in single and binary metal exposure experiments. *Environmental Pollution (Barking, Essex: 1987)*, *224*, 368‑376. https://doi.org/10.1016/j.envpol.2017.02.016
